# Do COVID-19 containment measures work? Evidence from Switzerland

**DOI:** 10.1186/s41937-022-00083-7

**Published:** 2022-02-05

**Authors:** Regina Pleninger, Sina Streicher, Jan-Egbert Sturm

**Affiliations:** 1 KOF Swiss Economic Institute, ETH Zurich, Leonhardstrasse 21, 8092 Zurich, Switzerland; 2 CESifo, Munich, Germany

**Keywords:** COVID-19, Reproduction rate, Stringency, Switzerland, H12, H51, H73, H75, I18, R59

## Abstract

We study the interplay of non-pharmaceutical containment measures, human behavior, and the spread of COVID-19 in Switzerland. First, we collect sub-national data and construct indices that capture the stringency of containment measures at the cantonal level. Second, we use a vector autoregressive model to analyze feedback effects between our variables of interest via structural impulse responses. Our results suggest that increases in the stringency of containment measures lead to a significant reduction in weekly infections as well as debit card transactions, which serve as a proxy for behavioral changes in the population. Furthermore, analyzing different policy measures individually shows that business closures, recommendations to work from home, and restrictions on gatherings have been particularly effective in containing the spread of COVID-19 in Switzerland. Finally, our findings indicate a sizeable voluntary reduction in debit card transactions in response to a positive infection shock.

## Introduction

The number of COVID-19 cases worldwide passed the 100 million mark at the end of January 2021. The number of deaths associated with the virus reached 4 million at the end of June 2021. The emergence of additional infection waves suggests that early removal of non-pharmaceutical containment measures may have had a huge impact on the number of cases and deaths. However, many governments are reluctant to take stronger measures due to economic concerns and public disapproval.

In this paper, we analyze the relationship between non-pharmaceutical containment measures, the spread of COVID-19, and public behavior in Switzerland. Compared to other European countries, Switzerland imposed, on average, less stringent measures despite being just as affected. In addition, Switzerland consists of 26 cantons, each of which enjoys extensive political autonomy. Especially the cantonal heterogeneity in the implementation of COVID-19-related containment measures provides an interesting environment to study the impact of mitigation measures. We exploit this cantonal variation to estimate the effects of containment measures on the spread of COVID-19.

The contribution of this paper is twofold. First, we collect cantonal data on non-pharmaceutical containment measures and construct an index capturing the stringency of these interventions. In particular, we closely follow the classifications used for the Oxford Stringency Index (Hale et al., 2020), but deviate in a number of dimensions to account for the Swiss setting.^1^ Second, we use the constructed indices to analyze the interplay between containment measures, public behavior, and the spread of COVID-19 in Switzerland using a vector autoregressive model (VAR) that accounts for the relationship of current and past observations of all variables in the system. In particular, our VAR model allows for feedback effects between containment measures, public behavior and the spread of COVID-19.

The results indicate that an increase in the stringency of non-pharmaceutical measures induces significant and sizable reductions in COVID-19 infection growth. A 10-unit increase in policy stringency results in a 34% reduction in weekly infections after six weeks. When considering different measures individually, we find that workplace and business closings as well as restrictions on gatherings are particularly effective in containing the spread of COVID-19. Further, stricter measures lead to a decrease in debit card transactions, which proxies behavioral changes in our model. A rise in infection growth leads to a policy reaction in the form of stricter containment measures by federal and cantonal governments. Similarly, the public reacts and decreases consumer spending. Our findings indicate that up to half of the reaction is voluntary.

**Fig. 1:**
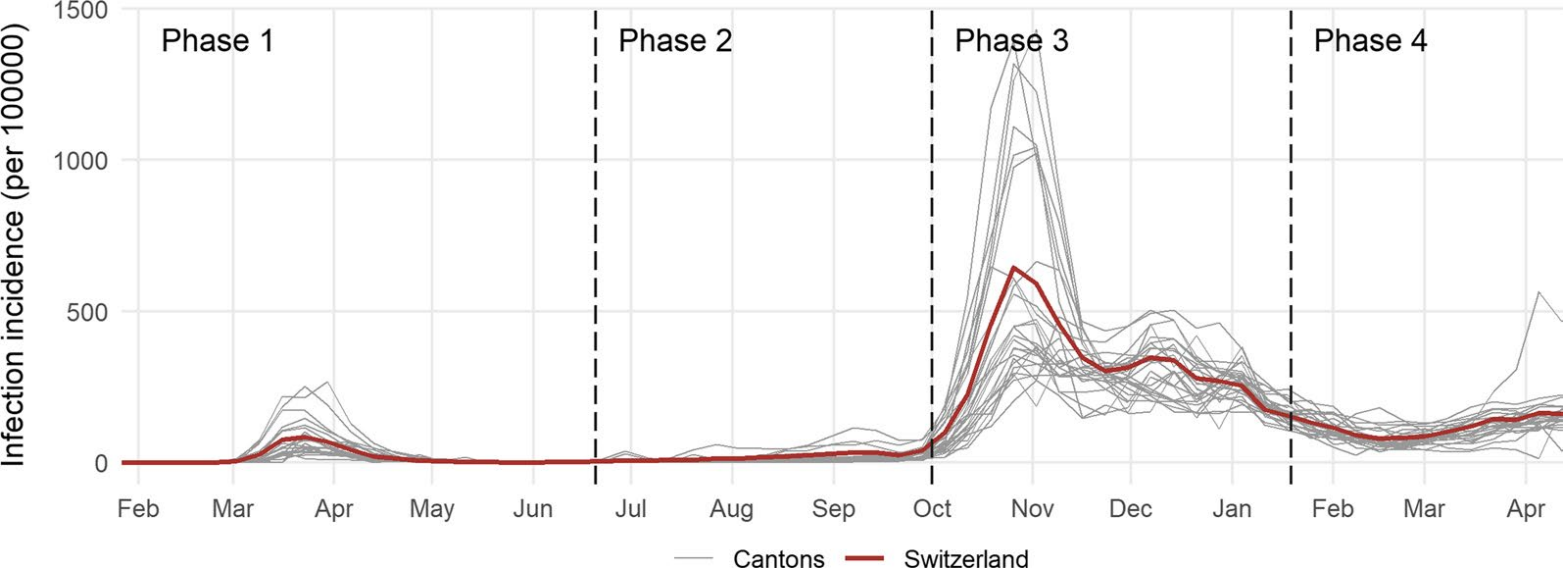
Weekly infection incidence. The weekly infection incidence reflects the number of confirmed cases per 100,000 residents during the respective week. The number of confirmed cases is provided by OpenZH (https://github.com/openZH/covid_19)

In our analysis, we divide the evolution of the pandemic in Switzerland into four phases. Phase 1 denotes the ‘extraordinary situation’ and spans from March 16, 2020, to June 19, 2020.^2^ During this first wave, the federal government mandated all COVID-19-related restrictions. Phase 2 begins after the extraordinary situation and ends with the termination of the federal ban on large-scale events on September 30, 2020. During this phase, case numbers were relatively low and many of the federal measures were relaxed, if not lifted. The third phase ranges from October 1, 2020, to January 17, 2021, and describes the second wave of the pandemic. Most cantonal variability is situated in this phase. Since June 20, 2020, the federal level effectively defined minimum non-pharmaceutical intervention measures and each canton decided for itself, depending on the local situation and its interpretation, to what extent it would go beyond these. This effectively ended on January 17, 2021, when the Federal Council implemented much more restrictive measures, thereby eliminating cantonal differences. Hence, the subsequent Phase 4 spans from January 18, 2021, to April 18, 2021, and is not only characterized by comprehensive federal restrictions, but also by the national vaccination campaign and the spread of the virus mutant B.1.1.7, nowadays called Alpha, first detected in the UK. On April 19, 2021, several policy relaxations became effective, such as the opening of restaurant terraces and indoor sport and cultural venues, marking the start of gradual easing. The evolution of the weekly infection incidence over the course of the four phases is shown in Fig. 1.

We limit our analysis to Phases 3 and 4 for multiple reasons. First, the effective reproductive number $R_e$, which we use to measure infection growth, only became available for all cantons by the end of March, thereby excluding the most important part of Phase 1. Additionally, the first wave constitutes an unexpected shock. The following phases are potentially quite different and more relevant for the future from a policy perspective. Moreover, the low level of infection incidence during Phase 2 entails high estimation uncertainty of $R_e$. More importantly though, such low levels of incidence likely suppress the reaction of policy and behavior to changes in infection growth. In contrast, for the remaining Phases 3 and 4, most of the time the 14-day incidence was far above a critical value of 60 and thus, the public and political awareness of the epidemiological situation was enhanced.

The analysis is relevant from a policy perspective. First, the effectiveness of containment measures has potential economic, social and political effects. This argument is particularly apparent as governments are often hesitant to impose stringent measures early on. Secondly, the results are important for potential future virus outbreaks. Even though the origin of COVID-19 is still under investigation, many former and current epidemics are zoonotic, that is, the disease spreads between animals and humans. The reduction in natural habitat and the increase in deforestation, urbanization, travel and mass food production is expected to increase the occurrence of viral outbreaks (Altizer et al., 2013). Last, once a viral disease emerges, additional mutations in response to natural or vaccine-elicited immunity pose a continued challenge to its containment. Hence, studying effective policy tools to circumvent future spreads early on is highly relevant from an economic, social, political and health perspective.

The next section presents some closely related literature on COVID-19 and other epidemics. We describe the empirical methodology in Sect. 3. Our KOF Stringency Indices and all other data are presented in Sect. 4. In Sect. 5, we present our findings. Section 6 concludes.

## Related literature

Although numerous studies have appeared since the outbreak of the COVID-19 pandemic in 2020, the literature that includes the additional waves of 2020/2021 is still sparse at the time of writing. Subsequent waves of infection provide additional insights and may be more representative for future outbreaks, as at least some level of preparation for further outbreaks has since been made. Before summarizing some relevant studies analyzing the COVID-19 pandemic, we first look into some based on the 1918 pandemic.

Hatchett et al. (2007) analyze non-pharmaceutical interventions (NPIs) in 17 U.S. cities during the 1918 influenza pandemic. They show that early interventions result in 50% lower peak death rates and less steep epidemic curves. The implemented interventions include closure of schools, churches and theaters. Similar results are reported by Bootsma and Ferguson (2007). They find a reduction in transmission rates of up to 30–50% in cities with comparably effective interventions, such as San Francisco, St. Louis, Milwaukee and Kansas City. However, the overall effect is only moderate, because—as they argue—measures were introduced too late or lifted too early. Related, Kremer (1996) shows that early public health interventions help to mitigate an unfavorable steady state in which the transmission of AIDS prevails. In addition, Kremer (1996) suggests that public health measures should target highly active people to reduce the number of partner changes.

Studies looking at the current crisis find that containment measures have a reducing effect on transmission rates, confirmed cases and deaths. Gatto et al. (2020) show that restrictions on mobility and human interactions led to a decrease in transmissions by 45% in Italy. Similarly, containment measures in China aiming at the protection of the susceptible population were particularly effective (Maier and Brockmann, 2020). In a cross-country study, Deb et al. (2020) report a reduction in the number of infections of up to 90% compared to the baseline scenario with no containment measures. In addition, they suggest that an immediate policy response significantly reduced the average number of cases and deaths. Huber and Langen (2020) find similar results for Switzerland. In particular, an earlier lockdown was more effective in reducing cumulative hospitalization and fatality rates. Caselli et al. (2020) suggest a reduction in the number of cumulative infections of up to 58% after 30 days of containment measures. The study also finds negative effects on confirmed cases after a period of 14 days. Similarly, Flaxman et al. (2020) suggest that around 3.1 million deaths have been averted until May 4, 2020, due to NPIs across 11 countries using a Bayesian hierarchical model. A related study by Brauner et al. (2021) analyzes the effects of individual NPIs. Their results suggest that school, university and face-to-face business closures as well as limits on gatherings were particularly effective. Chernozhukov et al. (2021) suggest that in the absence of business closures, the number of cases would have been 17–78% higher. Finally, Hsiang et al. (2020) show that anti-contagion policies significantly and substantially slowed infection growth. In particular, their results illustrate that early infections would have experienced exponential growth with growth rates of approximately 38% per day in the absence of policy actions. Bradley et al. (2021) calibrate an equilibrium model for the labor market in the presence of a pandemic in the UK and find that a laissez-faire approach implicate a higher death toll while total employment experiences a drop. The latter is, however, less pronounced than under a lockdown policy.

With regard to a reduction in case growth, Bendavid et al. (2021) find no significant benefits of stringent measures, including stay-at-home and business restrictions (‘lockdown’), compared to more lenient interventions, such as testing, bans on gatherings and other social distancing recommendations. However, Égert et al. (2020) and Acemoglu et al. (2020) suggest that selective containment measures that are targeted at the most vulnerable group in combination with increased testing lead to a reduction in deaths as well as economic losses.

For the USA, Gupta et al. (2020) find large declines in mobility in all states since the start of the COVID-19 pandemic. Yet a large part of the decline is not related to government policies, as mobility also fell in states without major restrictions and before any measures were implemented. Nevertheless, containment measures still have a significant effect on mobility reduction, where county policies had a larger impact than state policies. Both, Kraemer et al. (2020) and Tian et al. (2020) study mobility and travel restrictions during the Coronavirus pandemic in China. Kraemer et al. (2020) show that the spatial distribution of COVID-19 cases can be explained by human mobility data. Once mobility was restricted, the case growth turned negative. Related, the results in Tian et al. (2020) indicate that the Wuhan shutdown led to a delayed arrival of COVID-19 in other cities by almost three days, thereby limiting the spread of COVID-19 in China.

The effectiveness of government policies heavily depends on compliance by the population. This is particularly relevant for governments that focus on recommendations rather than stringent restrictions. At the same time, some measures are hard to enforce, since monitoring would constitute a violation of privacy. Therefore, when estimating the effects of non-pharmaceutical containment measures on COVID-19 infection rates, we include behavioral changes into the model. In the UK, Hacıoğlu-Hoke et al. (2021) find a drop in consumption even before lockdown measures were introduced. The decrease in expenditures was particularly pronounced in the top quartile of the income distributions.

## Methodology

In this section, we present our empirical approach to examine the interplay between COVID-19 infection growth (*I*), government containment policies (*P*), and behavior of the general population (*B*) in Switzerland. Changes in infection growth influence government policies and can lead to voluntary behavioral changes of the population. Policies are implemented to reduce infections and are often associated with far-reaching restrictions on citizens’ freedoms, leading to mandatory behavioral changes. Similarly, the behavior of the population has an effect on infection growth and, thus, on potential policies. As a consequence, all three variables affect each other.

A vector autoregressive model (VAR) constitutes a natural starting point for such an analysis. The identification of structural shocks from the reduced form representation using the Cholesky decomposition requires a specific ordering of the variables in the system. Given such an ordering, the first variable in the system does not depend on contemporaneous shocks to any other variable while the last variable is contemporaneously affected by all shocks. In our setting, such ordering is in fact sensible. The spread of the virus in a given week depends on the behavior of the population and the stringency of the policies in place that week. Behavior is to a high degree influenced by current policies. However, since data on infections are available with a considerable time lag ($k>0$), contemporaneous behavior depends only on past infection growth, $I_{t-k}$, where *t* denotes weeks. Determining measures to limit the spread of the virus usually requires negotiations between different ministries, parties, administrative levels, or at least within the Federal Council. Thus, once changes in the incidence of infection are observed, a response in the form of a policy change will not occur within the same week. The publication delay of information on infection growth further supports this argument. In contrast, since the exact impact of public behavior on infections is unknown to governments, we assume that they do not impose restrictions based on behavioral changes. Thus, policy changes do not directly depend on behavior, but are only indirectly affected through infection growth. In summary, we establish the following structural order:

1 $$\begin{aligned} P_t&= f\left( P_{t-1},\ldots ,I_{t-k},\ldots \right) \end{aligned}$$

2 $$\begin{aligned} B_t&= f\left( P_t, P_{t-1}, B_{t-1},\ldots ,I_{t-k},\ldots \right) \end{aligned}$$

3 $$\begin{aligned} I_t&= f\left( B_t, P_t, I_{t-1}, B_{t-1}, P_{t-1},\ldots \right) \end{aligned}$$

Additionally, we control for strictly exogenous variables regarding weather and holidays and a measure accounting for the vaccination progress.

Let $y_{i,t} {:}{=} \left( P_{i,t}, B_{i,t}, I_{i,t}\right) ',$ where *i* denotes the cantonal unit and let $x_{i,t}$ be an $r\times 1$ vector of contemporaneous control variables and cantonal fixed effects. The reduced-form ${\text {VAR}}(p)$ is given by

4 $$\begin{aligned} y_{i,t}&= \sum _{j=1}^p A_j y_{i,t-j} + C x_{i,t} + u_{i,t}, ~~~ u_{i,t} \overset{iid}{\sim } {\mathcal {N}}\left( 0, \Sigma _{u}\right) \end{aligned}$$

for $i = 1,\ldots ,n$ with ${\text {E}}\left[ u_{i,t} {u_{i,t}}'\right] = \Sigma _u$, where $u_{i,t}$ and $x_{i,t}$ are uncorrelated for all leads and lags. The $A_j$ are $3\times 3$ coefficient matrices and *C* is a $3\times r$ matrix. Since $\Sigma _u$ may have nonzero off-diagonal elements, the reduced-form error terms $u_{i,t}$ are likely correlated. Rewriting (4) in structural form by multiplying both sides by $B_0$ yields the structural error terms $w_{i,t}=B_0 u_{i,t},$ where $B_0^{-1}$ is the lower triangular Cholesky factor of $\Sigma _u$, i.e., $\Sigma _u = {B_0^{-1}}{B_0^{-1}}'$. Since $\Sigma _w ={\text {E}}\left[ w_t w_t'\right] = B_0 \Sigma _u B_0' = I_3$, where $I_3$ is the $3\times 3$ identity matrix, the structural errors $w_t$ are uncorrelated. The structural impulse responses (IR) are defined by

$$\begin{aligned} \frac{\partial y_{i,t}}{\partial w_{i,t-j}}, \end{aligned}$$

which can be obtained from the moving-average (MA) representation of (4), given by

$$\begin{aligned} y_{i,t} &= \sum _{j=0}^{\infty } \Phi _j C x_{i,t-j} + \sum _{j=0}^{\infty } \Phi _j u_{i,t-j} \\ &= \sum _{j=0}^{\infty } \Phi _j C x_{i,t-j} + \sum _{j=0}^{\infty } \Theta _j w_{i,t-j} \end{aligned}$$

with $\Phi \left( L\right) = \sum _{j=0}^\infty \Phi _jL^j = A\left( L\right) ^{-1}, A\left( L\right) = I_3-A_1L-\ldots -A_pL^p$ where $I_3$ is the $3\times 3$ identity matrix. Note that $A\left( L\right)$ is invertible given stationarity of $y_{i,t}$ (e.g., Kilian and Lütkepohl, 2017). The reduced-form impulse responses $\Phi _j$ can be retrieved recursively as $\Phi _0 = I_3, \Phi _j = \sum _{\ell =1}^j\Phi _{j-\ell }A_\ell$ for $j = 1,2,\ldots$ with $A_\ell =0$ for $\ell >p$ (Lütkepohl, 2005). Finally, the structural IRs are given by $\Theta _h = \Phi _h B_0^{-1}$ with $\Phi _0 = I_3$ and correspond to one standard deviation shocks to the three respective variables. To obtain standard errors, we use a wild bootstrap method detailed in Appendix 2.

### The effect of individual non-pharmaceutical interventions

The approach detailed above uses a composite measure of policy stringency (see Sect. 4), and thus, the method does not quantify the effectiveness of specific containment measures. Given that policy makers usually pass a package of different measures, disentangling the effects of specific measures is only possible if there is sufficient cross-sectional variation as well as variation over time. To enable the analysis of stringency sub-categories, we rely on a local projection (LP) approach.

Given that $y_t$ and $x_t$ are stationary, a VAR specification with infinitely many lags gives the same impulse response functions as a local projection approach (Jordà, 2005; Plagborg-Møller and Wolf, 2021) that accounts for the given ordering. Let ${\tilde{\upsilon }}_{\ell ,t} {:}{=} 100\ \frac {\upsilon _{\ell ,t}}{N_j}$ be the normalized policy value for category $\ell \in L,$ and $P_{-L,t}$ the policy stringency index computed with the remaining sub-categories. Obtaining LP impulse responses involves regressing the endogenous variable of interest on a set of contemporaneous exogenous control variables and lagged endogenous variables. This is done for each forecast horizon separately. To that end, the dependent variable is shifted forward corresponding to the forecast horizon $h=0,\ldots ,H$. The local-linear projection equations are given by

5 $$\begin{aligned} \begin{aligned} I_{i,t+h}&= \gamma ^h x_{i, t} + \sum _{j=0}^{p} \sum _{\ell \in L} a_{{\tilde{\upsilon }}_\ell ,j}^h {\tilde{\upsilon }}_{\ell ,i,t-j} + \sum _{j=0}^{p} a_{IP,j}^h P_{-L,i,t-j} \\&~~~~~+ \sum _{j=0}^{p} a_{IB,j}^h B_{i,t-j} + \sum _{j=1}^{p} a_{II,j}^h I_{t-j} + \epsilon _{I,i,t+h}^h \end{aligned} \end{aligned}$$

for each horizon $h=0,\ldots ,H.$ The LP-IRs are defined by ${\text {E}}\left[ I_{t+h} \big | \epsilon _{\cdot ,t} = 1, x_t, P_t, B_t, \ldots \right] - {\text {E}}\left[ I_{t+h} \big | \epsilon _{\cdot ,t} = 0, x_t, P_t, B_t, \ldots \right]$ for $h\ge 0$ and correspond to one-unit shocks. Thus, for sub-category $\ell$ and remainder index $P_{-L}$ the LP-IRs are given by $a_{{\tilde{\upsilon }}_\ell ,0}^h$ and $a_{IP,0}^h$, respectively (Plagborg-Møller and Wolf, 2021).^3^ The cumulative IRs can be obtained by replacing the left hand side of (5) with $\sum _{{\tilde{h}}=0}^h I_{i,t+{\tilde{h}}}$.

## Data

We approximate our three endogenous variables *P*, *B*, and *I* by the KOF Stringency-Plus Index (${\text {KSI}}^+$), consumption captured by the number of debit card transactions (NTRX) and new infections (NINF), respectively. To ensure stationarity, we use first differences or log-differences. In particular, we measure the policy responses by the difference in the KOF Stringency-Plus Index $P_{i,t} = \Delta {\text {KSI}}_{i,t}^+$ and consumer spending by the weekly growth in the number of domestic debit card transactions, i.e., $B_{i,t}=\Delta \ln {\text {NTRX}}_{i,t}$. Last, infection growth is approximated by the logarithm of the effective reproductive number $I_{i,t} = \ln R_{e,i,t} \approx \Delta \ln {\text {NINF}}_{i,t}$ (see Appendix 3). To facilitate notation, we continue to use *P*, *B* and *I*. We first present our indices capturing non-pharmaceutical interventions and subsequently discuss our infection and behavior variables as well as all exogenous control variables.

### Policy (P): KOF stringency indices

The KOF Stringency Index (${\text {KSI}}$) and KOF Stringency-Plus Index (${\text {KSI}}^+$) record the stringency of COVID-19 containment measures in Switzerland. The indices are composite measures including different lockdown policies, such as school and workplace closures, restrictions on gatherings, and travel restrictions. The values range from 0 (= no measures) to 100 (= full lockdown). Both indices build upon the coding framework of the Oxford Stringency Index (Hale et al., 2020).

Despite the existence of cantonal differences, the Oxford Stringency index for Switzerland is only available at the national level. Moreover, the (national) Oxford Stringency index not only reflects national decisions, but also regional measures if they are more stringent than the national ones. In order to account for their regional relevance, cantonal measures receive less weight. Thus, the index neither necessarily reflects nation-wide restrictions, nor allows for a regional interpretation. The KOF Stringency Indices close these gaps. For the aggregate index, only nation-wide measures are included while the cantonal indices also reflect all canton-specific restrictions. These indices allow for a comparison between cantons as well as between national and cantonal stringency levels. Since cantons are obliged to implement the national measures but can introduce stricter measures if preferred, the national index, in general, constitutes a lower bound for the canton-specific indices. Only between December 11, 2020, and January 9, 2021, cantons were able to deviate from this rule provided their effective reproductive number ($R_e$) remained below 1.0 and the weekly incidence below the Swiss average for at least seven days.^4^

The construction of the KOF Stringency index (${\text {KSI}}$) closely resembles Oxford’s stringency index. In particular, it is given by the normalized sum of all stringency sub-categories, i.e.,

6 $$\begin{aligned} {\text {KSI}} = \frac{1}{9} \sum _{j=1}^{9} \left( 100 \cdot \frac{\upsilon _{j,t}}{N_j}\right) , \end{aligned}$$

where $\upsilon _{j,t}$ is the policy value for sub-indicator *j* on day *t* and $N_j$ its maximum value. The KOF and Oxford Stringency indices consist of nine sub-indicators, namely school closing, workplace closing, cancellation of public events, restrictions on gatherings, closure of public transport, stay-at-home requirements, restrictions on internal movement, international travel controls and public info campaigns. The coding of these sub-indicators is identical to that of the components of the Oxford Stringency Index.^5^

For the KOF Stringency-Plus Index, we adapt the original KOF Stringency Index along two dimensions. First, we include facial coverings as an additional sub-indicator. This variable is also collected by the Oxford Covid-19 Government Response Tracker and used to construct additional indices. Second, we transform the sub-indicator related to restrictions on workplaces (*c2_workplaceclosing*) by adding another category that accounts for the reduction in opening hours and capacity.^6^ Thereby, we are able to incorporate restaurant policies more precisely than in the original stringency index. Using these ten sub-indicators, the formula above changes to:

7 $$\begin{aligned} {\text {KSI}}^+ = \frac{1}{10} \sum _{j=1}^{10} \left( 100 \cdot \frac{\upsilon _{j,t}}{N_j}\right) , \end{aligned}$$

where $\upsilon _{j,t}$ is the policy value and $N_j$ is the maximum possible value for sub-indicator *j*.

**Fig. 2:**
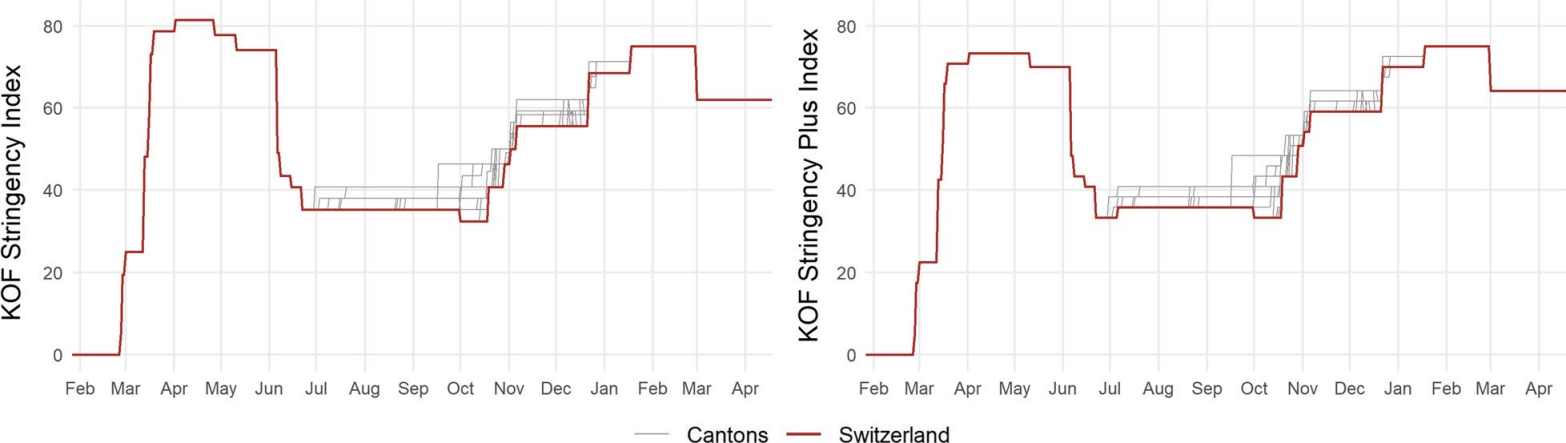
KOF stringency indices for Swiss cantons. The graph depicts the KOF Stringency Index (left) and the KOF Stringency-Plus Index (right). The respective index is denoted on the *y*-axis. Note that cantonal variation only starts at the end of June. The first lockdown was governed by federal measures

We collect data for each sub-indicator from a variety of sources (see source list in Appendix 1) and calculate the KOF Stringency Index and KOF Stringency-Plus Index for Switzerland and all of its 26 cantons. Figure 2 shows the evolution of both indices over time. On March 16, 2020, the Swiss Federal Council declared the ‘extraordinary situation’ in terms of the Epidemics Act and enforced far-reaching national containment measures. As cantons were obliged to implement all national measures, no cantonal variation existed until mid-June, 2020. On June 19, 2020, the ‘extraordinary situation’ ended and from then on, federal measures constituted minimal restrictions for cantonal governments, which were able to impose stronger measures if considered necessary. This opportunity to act at the cantonal level was in particular used in the French-speaking part of Switzerland. Figure 2 shows substantial cantonal variation after the end of the extraordinary situation, especially during fall and winter.

**Fig. 3:**
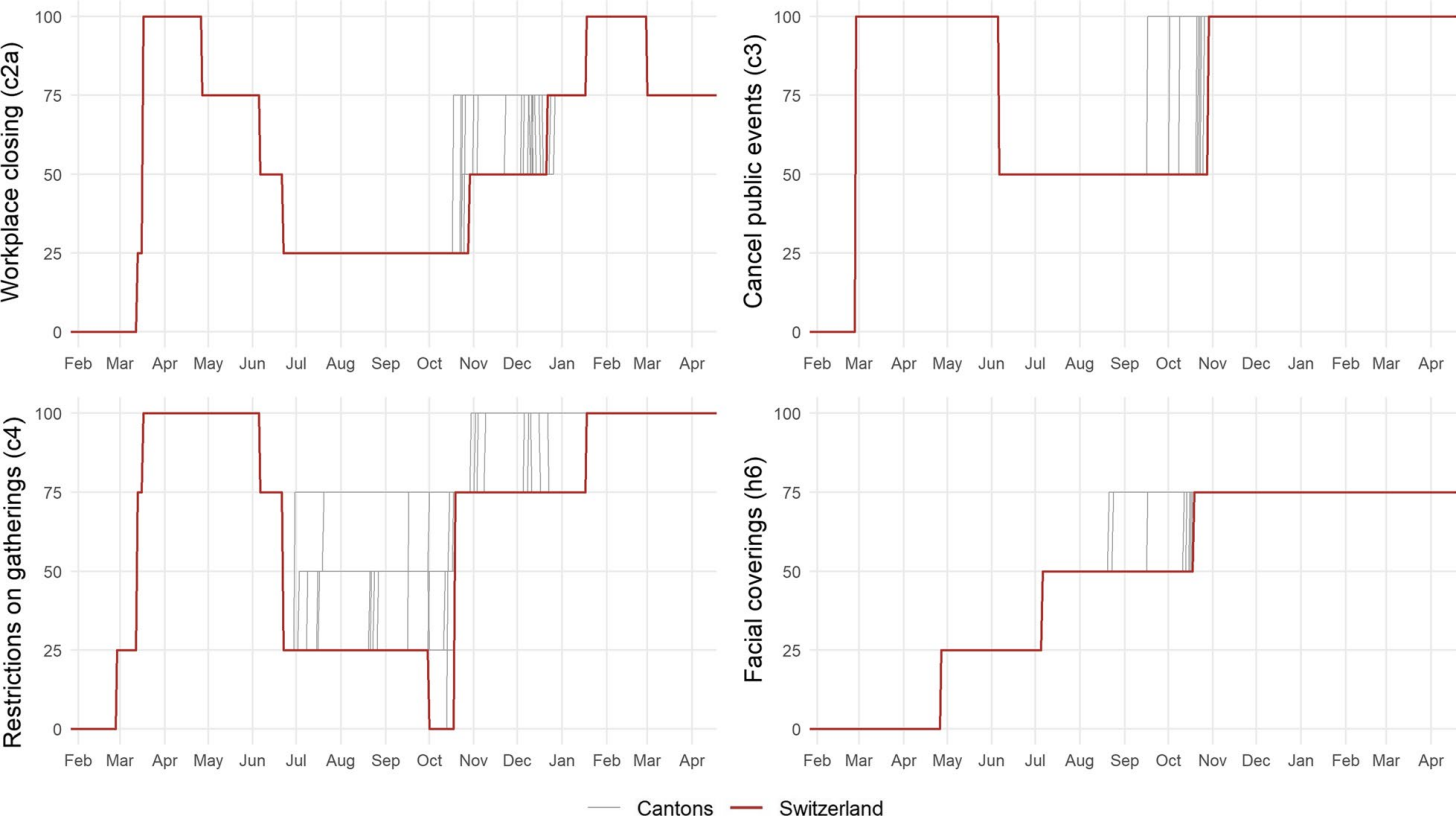
Sub-indicators of the ${\text {KSI}}^+$with cantonal variation. The graph shows the sub-indicators of the KOF Stringency-Plus Index that exhibit cantonal variation. The respective sub-indicator is denoted on the *y*-axis. Note that cantonal variation only starts at the end of June. The first lockdown was governed by federal measures

Figure 3 depicts the sub-categories of the KOF Stringency-Plus Index (${\text {KSI}}^+$) that vary over time and across cantons. The other categories are provided in Fig. 12 in Appendix 4. The largest variation is observed for restrictions on gatherings (bottom left of Fig. 3). On 1 October 2020, the federal government withdrew the restrictions on gatherings and increased the autonomy of cantonal governments to impose restrictions they deem necessary in their localities. Consequently, restrictions on gatherings returns to zero. From the end of April, the use of facial coverings was phased in over the remainder of 2020.

The category workplace closing (top left panel of Fig. 3) provides another interesting insight. There are a number of cantons that closed restaurants and businesses in October 2020. These cantons are mainly from the French-speaking part of Switzerland. In contrast, workplace closures in December 2020 were driven by individual German-speaking cantons and later by the federal government.

### Infection growth (I): effective reproduction number

We measure the spread of the virus by the effective reproductive number $R_e$ based on newly confirmed cases. It represents the number of secondary infections caused by a previously infected individual. Whenever $R_e$ is above one, the number of new infections increases exponentially, while for $R_e$ below one, the spread of the virus decreases. We use $R_e$ provided by Huisman et al. (2020), who estimate $R_e$ for all cantons of Switzerland. To that end, they first smooth the series of newly confirmed cases by local polynomial regression fitting (LOESS) to cope with reporting cycles and irregular reporting practices.^7^ In a next step, they deploy a deconvolution step using suitable delay distributions between transmission and reporting to infer the infection incidence.^8^ Last, Huisman et al. (2020) use the EpiEpstim method developed by Cori et al. (2013) to estimate $R_e$ from the series of infection incidence.^9^

**Fig. 4:**
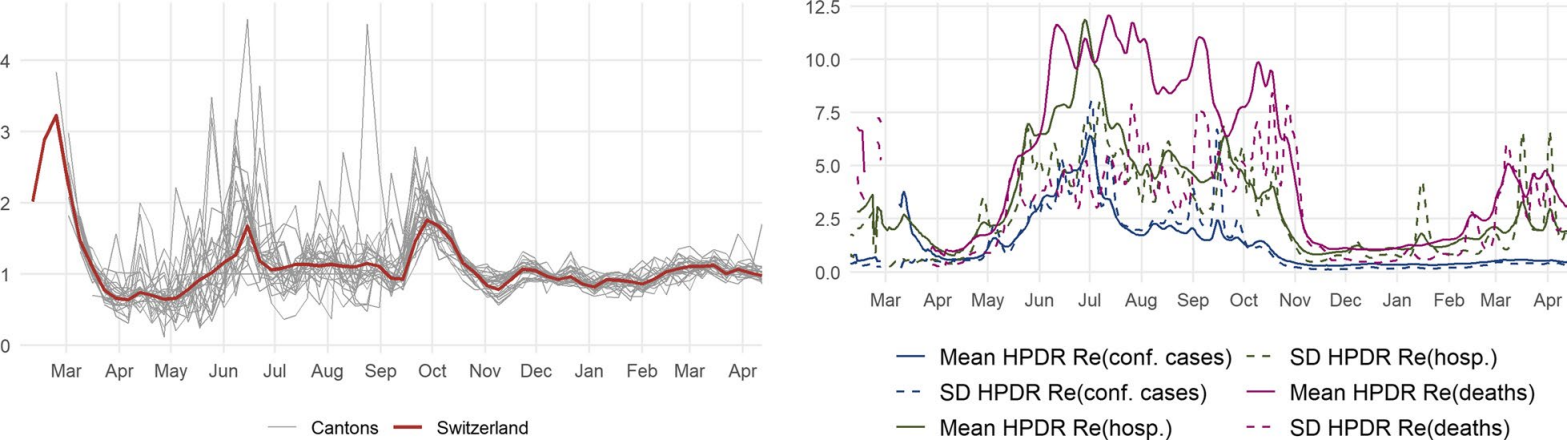
Effective reproduction number $R_e$ and estimation uncertainty. The effective reproduction number $R_e$ (left panel) is estimated by Huisman et al. (2020) using the EpiEstim method by Cori et al. (2013). The right panel shows the uncertainty associated with the estimation of $R_e$ based on confirmed cases, hospitalizations and deaths. The solid (dashed) lines represent the daily mean (standard deviation) of the highest posterior density range (HPDR) across cantons

The weekly averages of the cantonal $R_e$s based on confirmed cases are shown in the left panel of Fig. 4 and the weekly infection incidence in Fig. 1. The weekly infection incidence reflects the number of newly confirmed cases within that week per 100,000 residents. In 2020, the level of daily infections was particularly high from March until May and from October onward. The difference between these two phases can in part be attributed to limited testing capacities during the first wave of infections compared to the second starting in the fall. Correspondingly, during the early months of the pandemic, $R_e$ reached values of above 3 in many cantons, which, combined with the high level of daily infections, resulted in far-reaching containment measures, subsequently pushing $R_e$ below one. During the summer, $R_e$ mostly fluctuated around one and occasionally peaked in some cantons. Due to the generally low level of infections, these increases were easily contained.

$R_e$ started rising again in mid-September, triggering more stringent policy restrictions. Since October 2020, the cantonal reproduction rates appear to have moved more in tandem, hovering around 1. The right panel of Fig. 4 shows the uncertainty involved in the estimation of $R_e$. It reports the mean and standard deviation of the highest posterior density range (HPDR) across all cantons for $R_e$ based on confirmed cases (blue), hospitalizations (green), and deaths (purple). During the summer, when there were few confirmed infections and, thus, even fewer hospitalizations and deaths, uncertainty increased considerably. We use $R_e$ based on confirmed cases, as its level of uncertainty appears relatively stable compared to the two alternatives.

One advantage of $R_e$ as a measure for infection growth is its reflection of local transmissions (Huisman et al., 2020). Cases that were imported from abroad (but tested positive in Switzerland) are neglected to avoid distortions of domestic transmission developments. Only cases stemming from infections in Switzerland are included. Another benefit of $R_e$, compared to confirmed cases growth, is the consideration of the susceptible population, which is the fraction of population that is not yet immune. Most importantly, using $R_e$ as a measure of infection growth mitigates possible endogeneity problems. Changes in infection dynamics most likely affect government decisions on containment measures, thus affecting the KOF Stringency Indices. Similarly, rising infection growth increases the risk of getting infected, possibly triggering a voluntary reduction in consumer spending and other behavioral changes. Given that cantonal $R_e$ is available with a delay of 14 to 17 days, i.e., in real time there is quite some uncertainty about current reproduction rates, $R_e$ effectively affects stringency measures and mobility with a considerable (publication) delay.

### Behavior (B): debit card transactions

**Fig. 5:**
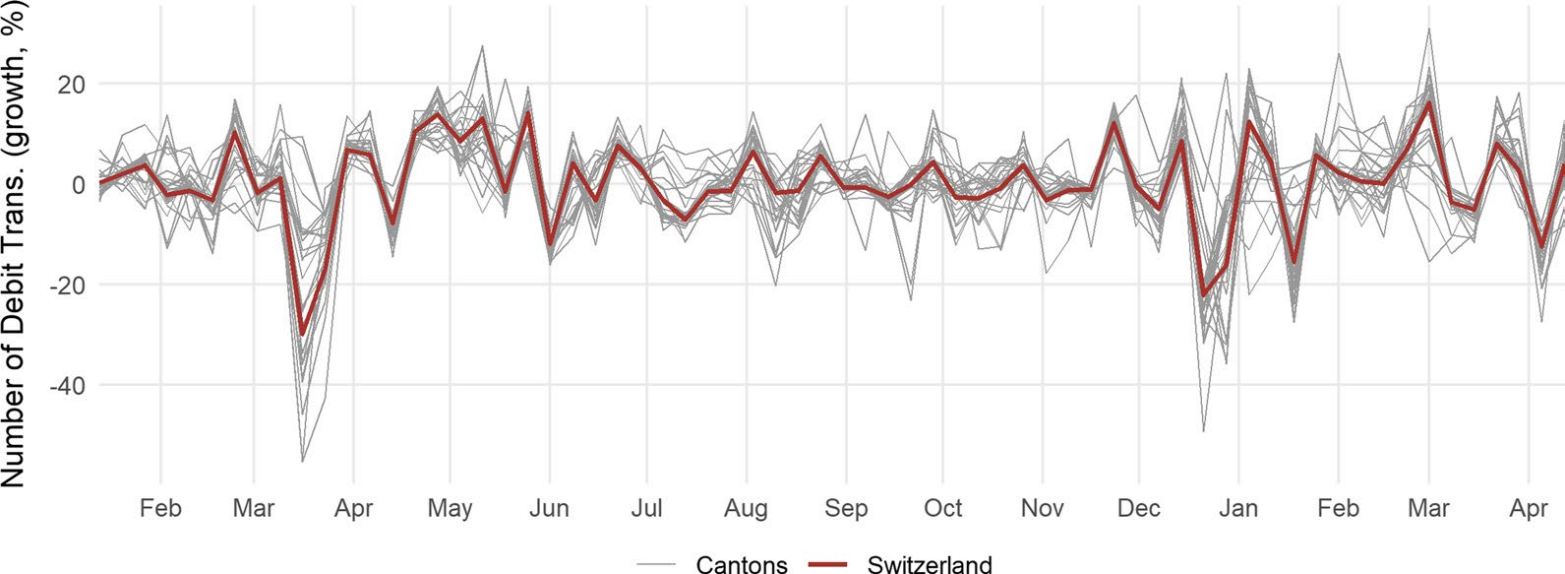
Number of debit card transactions. The number of debit card transactions reflects the number of transactions in each canton made by Swiss debit card holders. The data span January 1, 2020, until April 18, 2021. The weekly growth rate is given in per cent

Containment measures related to non-essential retail business closures or restaurant lead to declines in mobility as well as spending. To measure household spending, we use the number of transactions in CHF by Swiss debit card owners, provided by SIX BBS AG through Monitoring Consumption Switzerland.^10^ We exclude all ATM transactions as they are subject to monthly seasonality. Figure 5 displays the daily growth rate (in %) of the number of Swiss debit card transactions in each canton. In mid-March 2020, when the first lockdown was enacted, there was a sizable reduction in spending. Similarly, at the end of December 2020, with the start of the Christmas holidays and the national reintroduction of restaurant closures, consumer spending decreased considerably.

Consumption can be seen as a measure for the level of social distancing by the population. Less spending implies that fewer potentially infected individuals come into contact with non-infected ones.^11^ Changes in the number of debit transactions thus represent changes in behavior, possibly due to containment measures, but also as an individual response to the level of infection growth.

**Fig. 6:**
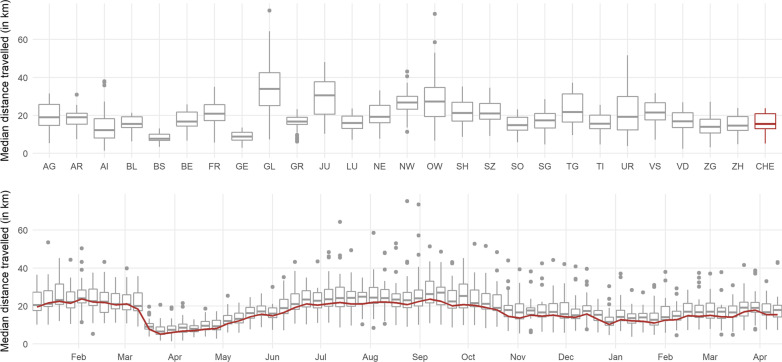
Mobility. Mobility is measured as the median distance in kilometers travelled by a sample of tracked cell phone users. The upper and lower parts show box plots grouped at the canton level and across cantons at the weekly level, respectively. National figures are shown in red. The data span January 1, 2020, until April 18, 2021

Similarly, changes in behavior can be quantified by changes in mobility. On behalf of StatisticsZH, the Swiss National COVID-19 Science Task Force, and the KOF Swiss Economic Institute, intervista AG publishes daily mobility data for a representative sample of the Swiss population based on smartphone movement data. We use the daily median distance measured in kilometers and take weekly averages, see Fig. 6. The reduction in mobility starting mid-March, after strict containment measures were imposed, is clearly visible. However, the decline in mobility was less pronounced in the fall, when infection rates started rising and containment measures were enacted again.

In our main analysis, we use the number of debit card transactions to measure the behavior of the population. The underlying data set on debit card transaction data is comprehensive in that it includes all transactions conducted in Switzerland, broken down by canton. In contrast, the data set on mobility consists of 2500 individuals in Switzerland. As a result, the mobility data are not necessarily representative for each canton. Nevertheless, we conduct robustness checks using mobility to proxy behavior.

### Other data: control variables

The likelihood of infection depends on the extent of social distancing. The number of daily contacts naturally changes when there is a break in daily routines, for instance during school or public holidays. For that reason, we build daily indicators reflecting school holidays in each canton based on information provided by the Swiss Conference of Cantonal Ministers of Education. If all schools in a canton are on holiday, the indicator is set to one, while for holidays that only affect a part of a canton, the indicator is set to 0.5. Additionally, we incorporate a dummy variable that reflects cantonal as well as federal public holidays. The underlying data were obtained via manual web-scraping. To obtain weekly indicators, we take averages.

Weather conditions could also have an influence on the behavior of citizens and the contagiousness of the virus.^12^ We rely on the MeteoSwiss reference monitoring network SwissMetNet (SMN), provided by the Federal Department of Home Affairs (FDHA), to construct weather variables for each canton. Specifically, we use the maximum daily temperature in degree Celsius, daily precipitation in millimeters, daily sunshine hours, and daily mean relative humidity. The SMN consists of approximately 160 automatic weather monitoring stations. We match each SME station with a Swiss municipality and subsequently compute population weighted versions of all weather variables for each canton. The municipal population figures reflect the permanent resident population on December 31, 2019, provided by the Swiss Federal Statistical Office (SFSO). This procedure excludes highly elevated mountain stations and ensures that the cantonal weather variables reflect the weather in populous regions. For the cantons Appenzell-Innerrhoden (AI), Appenzell-Ausserrhoden (AR), Basel-Stadt (BS), and Nidwalden (NW), no station matches were found. Taking their location into account, we approximate AI and AR by St. Gallen (SG), BS by Basel-Landschaft (BL), and NW by Obwalden (OW). Finally, we produce weekly averages for all variables. Summary Statistics of all variables are provided in Table 1.

Finally, we want to account for progress in vaccination rates during the period under investigation. The first dose was administered on December 23, 2020, and due to availability and administrative constraints, the vaccination campaign in Switzerland prioritizes the elderly population and those that are in close contact with them until May 2021. Even though the vaccination roll-out was mainly driven by supply-side constraints in the beginning, the actual cantonal vaccination progress might be endogenous. To circumvent this problem, we interact the nationwide vaccination progress with the cantonal share of the population of age 65 and older. For the vaccination progress, we consider both the newly partially and newly fully vaccinated. These two interaction terms capture cantonal heterogeneity, are not endogenous to changes in infection rates and the average correlation to the cantonal vaccination progress is large with 0.82 (0.87) for the partially (fully) vaccinated during our estimation period.

**Table 1:** Summary statistics

	Mean	Sd	Min.	1st Qu.	Median	3rd Qu.	Max.
$\Delta$ KOF Stringency-Plus Index	0.917	3.550	− 10.833	0	0	1.190	13.333
$\Delta \ln$ Number of debit card transactions	0.001	0.100	− 0.493	− 0.043	0.005	0.058	0.312
ln effective reproductive number	0.027	0.213	− 0.573	− 0.100	0.007	0.110	0.974
$\Delta$ median distance (in km)	− 0.168	4.910	− 33.296	− 1.975	− 0.105	1.855	33.119
Public holiday	0.031	0.077	0	0	0	0	0.286
School holiday	0.279	0.401	0	0	0	0.500	1
$\Delta$ maximum temperature (in °C)	− 0.297	4.566	− 12.283	− 3.471	− 0.773	2.506	15.047
$\Delta$ precipitation (in mm)	− 0.203	4.343	− 27.312	− 2.227	− 0.474	1.556	16.943
$\Delta$ sunshine hours	0.101	2.042	− 5.686	− 1.302	− 0.001	1.428	6.492
$\Delta$ relative humidity (in %)	− 0.434	7.517	− 24.086	− 5.528	0.194	4.597	23.155
$\Delta$ school closing (c1)	1.149	6.086	0	0	0	0	33.333
$\Delta$ workplace closing (c2a)	1.724	8.953	− 25	0	0	0	50
$\Delta$ cancel public events (c3)	1.658	6.483	0	0	0	0	50
$\Delta$ restrictions on gatherings (c4)	2.155	11.699	− 17.857	0	0	0	75
$\Delta$ close public transport (c5)	1.724	6.408	0	0	0	0	28.571
$\Delta$ stay at home requirements (c6)	0	8.206	− 33.333	0	0	0	28.571
$\Delta$ domestic travel (c7)	0	12.309	− 50	0	0	0	42.857
$\Delta$ international travel (c8)	0	0	0	0	0	0	0
$\Delta$ public info campaign (h1)	0	0	0	0	0	0	0
$\Delta$ facial coverings (h6)	0.763	4.067	0	0	0	0	25
Incidence	220.927	187.279	7.650	104.431	167.691	291.886	1432.723
$\Delta share_{\ge 65}~\cdot$ persons fully vaccinated (CH)	1.609	2.017	0	0	0.001	3.575	6.292
$\Delta share_{\ge 65}~\cdot$ persons partially vaccinated (CH)	1.200	2.339	− 2.925	0	0	2.962	8.132

The sample includes weekly data from September 28, 2020, until April 18, 2021, for each of the 26 cantons, amounting to $N=754$ weeks. The stringency sub-categories are normalized by their respective maximum value and multiplied with 100. The infection incidence reflects the number of confirmed cases per 100,000 residents during the respective week. $\Delta$ denotes the first difference operator and ln the natural logarithm

## Results

All models are estimated using ordinary least-squares (OLS) with weekly data and four lags ($p=4$).^13^ Regarding the information delay of the effective reproductive number $R_e$, we assume $k=3$, i.e., there is no effect of changes in infection growth for two weeks, which is in accordance with the publication lag of 14–17 days. Note that the underlying variables for *B* and *I* enter the model in log differences while the one for *P* and all weather-related variables enter in first differences. Our estimation sample covers Phases 3 and 4 which amounts to $T=29$. We exclude Phases 1 and 2 for four reasons. First, the effective reproductive number $R_e$ only became available for all cantons by the end of March, which given our lag structure eliminates the most important part of this phase. Second, since the first wave constitutes an unexpected shock, it is potentially quite different and less relevant for the future from a policy perspective. Third, during Phase 2, the incidence level was very low or even zero in some cantons, which entails high estimation uncertainty of the reproductive number $R_e$ (see Fig. 4). Lastly, the low level of incidence during Phase 2 likely suppresses the reaction of policy and behavior to changes in infection growth. In contrast, all through Phases 3 and 4, the 14-day incidence was close to but most of the time far above the critical value of 60, as specified by the Federal Office of Public Health FOPH.

In what follows, we focus on the analysis of cumulative impulse response (IR) functions. In contrast to regular IRs, which show the response of the involved variables as they enter the model, i.e., in log-differences or first differences, the cumulative IRs provide the reaction of the underlying variables in (log-) levels. For instance, the cumulative IR at horizon *h* of infection growth $I=\Delta \ln R_e$ to a shock to policy $P=\Delta$ ${\text {KSI}}^+$ shows the change between $\ln {\text {NINF}}_{t+h}$ and $\ln {\text {NINF}}_{t-1}$, which corresponds to the *h*-week growth rate of the number of new infections (NINF), see Appendix 3. Hence, $100\cdot \left( \exp \left\{ s \cdot {\text {IR}}_h^{cum} \right\} -1\right)$ percentage points are the response of the level of new infections, where *s* is the size of the policy shock and ${\text {IR}}_h^{cum}$ the cumulative IR at horizon *h* to a unit shock.^14^

### Interplay between infections (*I*), behavior (*B*) and policy (*P*)

We estimate model (4) with and without time fixed effects.^15^ As it brings us closer to estimating causal relationships, our analysis will concentrate on the model that contains time fixed effects. When time fixed effects are included, all changes relevant for all cantons to the same extent are absorbed and the estimated coefficients represent the effects of the cantonal changes in the involved variables. For instance, national policy changes are not reflected in the estimated parameters. The same applies to co-movement in $R_e$ or consumption. Hence, the impulse responses of the model with time fixed effects correspond to marginal changes in the involved variables. The time fixed effects do, however, ensure that any time-period specific effects not captured by the included variables do not distort our analysis. Henceforth, we call this model the canton-specific model. In contrast, when time fixed effects are excluded, the estimated coefficients incorporate all cantonal and other changes. Due to possibly omitted time-specific effects, the difference between the two models might account for more than just changes at the national level, such as infection rates and containment measures in neighboring countries. Nevertheless, we include this model in our analysis as it offers a useful comparison to, and validation of, the more restrictive model with time fixed effects. Henceforth, we call the model without time fixed effects the combined model.

**Fig. 7:**
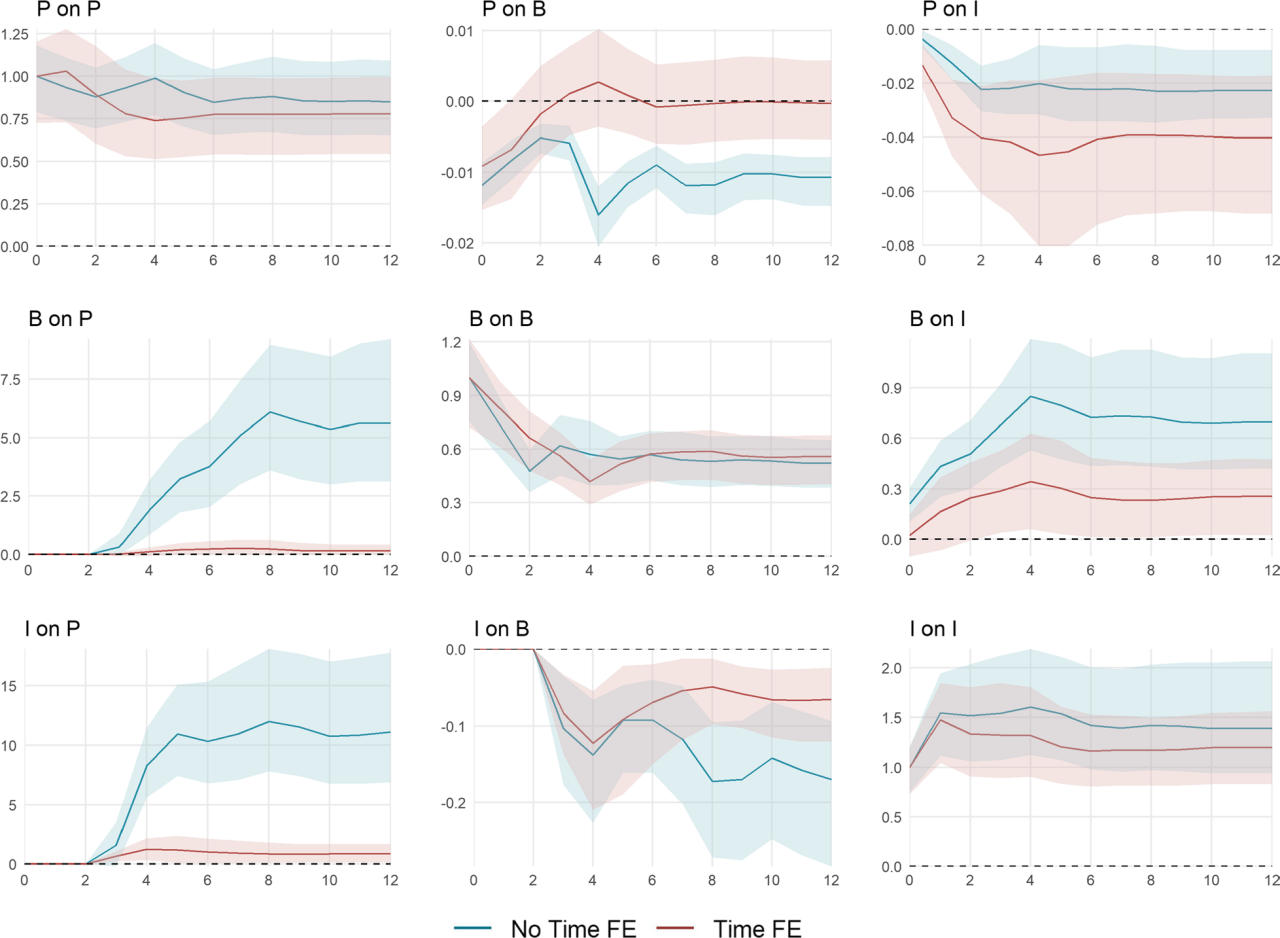
Cumulative impulse responses of policy *P*, behavior (consumption) *B*, and infection growth *I*. The impulse responses are estimated using the recursively ordered VAR(4) in (4) with $k=3$ and $P=\Delta$ ${\text {KSI}}^+$, $B=\Delta \ln {\text {NTRX}}$, and $I = \ln R_e$ with (red) and without (blue) time fixed effects. The data spans September 28, 2020, until April 18, 2021. The shaded areas represent the 95% confidence intervals based on a wild bootstrap procedure with 5000 repetitions. The horizontal axes depict the time horizon in weeks. The vertical axes show the level response of the ${\text {KSI}}^+$ (left), $\ln {\text {NTRX}}$ (middle), $\ln {\text {NINF}}$ (right)

The cumulative impulse responses implied by the estimation of (4) with (red) and without (blue) time fixed effects are shown in Fig. 7.^16^ They are standardized to allow for an easy comparison between both models. The top panels show the cumulative impulse responses to a policy shock, the middle panel those to a behavior shock, and the bottom panel those to a shock to infection growth. The horizontal axes depict the time horizon in weeks. The vertical axes show the level response of the ${\text {KSI}}^+$ (left), $\ln {\text {NTRX}}$ (middle), $\ln {\text {NINF}}$ (right).

A one-unit policy shock induces a permanent increase in the ${\text {KSI}}^+$. In the canton-specific model, policy is partially withdrawn in the subsequent weeks. In the combined model, the level of transactions is permanently decreased after a policy increase. In contrast, the canton-specific shocks significantly reduce debit card spending for only two weeks by about − 1%. This difference traces back to the difference in the policy response itself: After four weeks, policy is tightened anew, resulting in a stronger reduction of approximately − 1.6%. The effect of policy on the level of infections appears somewhat stronger in the canton-specific model, however, horizon one and two are the only horizons for which the impulse responses significantly differ from each other. In the model with time fixed effects, after six weeks, a 10-unit increase in the ${\text {KSI}}^+$ leads to a $\exp (-0.041\cdot 10)-1=-34\%$ decrease in the level of weekly infections.

A shock in debit card transactions permanently increases the level of transactions. By construction, behavior only has an impact on policy through infection growth and, thus, this effect is only significant for the combined model. A ten-percentage points shock of debit transaction growth leads to a $3.78\cdot \ln \left( 1.1\right) = 0.36$ unit increase in the ${\text {KSI}}^+$ after six weeks. A positive transaction shock also has a positive effect on infection growth. A ten percent shock increases the level of new weekly infections by $1.1^{0.21}-1=2.0\%$ on impact and by $1.1^{0.85}-1=8.4\%$ after four weeks. The canton-specific effect is generally less pronounced. This indicates diminishing marginal costs of behavior on infections.

An infection shock induces a permanent level shift in the number of new infections. A ten percent increase approximately leads to a $1.1^{1.5}-1=15\%$ rise during four subsequent weeks. For the combined model, the same shock implies a $8.3\cdot \ln \left( 1.1\right) = 0.79$ unit increase in the ${\text {KSI}}^+$ after four weeks, while marginal cantonal policy only increases by $1.3\cdot \ln \left( 1.1\right) = 0.12$ units. Accordingly, and similar to the behavioral reaction to policy, the effect of behavior to an infection shock is less pronounced in the canton-specific model. This is partly due to the comparably small marginal policy reaction. Additionally, the cantonal non-pharmaceutical measures could be less targeted at limiting virus spread through a reduction in overall consumption. The overall decrease in debit card spending after a 10 percent increase in the number of new infections amounts to about $1.1^{-0.13}-1=-1.2\%$ at a horizon of four weeks. The canton-specific effect becomes significantly different from week 8 onward.

The estimation results for both models are shown in Tables 2 and 3 in Appendix 5. In the combined model without time fixed effects, public and school holidays and increases in the interaction between the share of the population that is 65 years and older ($share_{\ge 65}$) with the newly fully vaccinated decrease the number of debit transactions. Rising temperature and the increases in the number of newly partially vaccinated (interacted with $share_{\ge 65}$) lead to an increase. Moreover, holidays are positively associated with the introduction of NPIs and public holidays with infection growth. Though not statistically significant, increasing temperature, sunshine hours and relative humidity are negatively and precipitation positively related to infection growth. When time fixed effects are included, all connections between the exogenous variables and changes in the ${\text {KSI}}^+$ turn insignificant. The relationships with debit card transactions remain largely the same. While the coefficient on relative humidity and precipitation is now significant and negative, that with vaccinations (interacted with $share_{\ge 65}$) turns insignificant. School holidays and infection growth are positively related. The newly partially vaccinated persons (interacted with $share_{\ge 65}$) show a negative relation to infection growth.

### Direct effects

The impulse responses presented in Sect. 5.1 represent all direct and indirect effects in the estimated VAR system. In this section, we want to disentangle direct from indirect effects by artificially implementing zero restrictions on the respective indirect transmission channels, before recomputing the impulse response functions. To be more precise, to compute the direct effect of variable *m* on variable *n*, we set $a_{j,nq}=0$ for $j=1,\ldots ,p$ and $q = \left\{ 1,2,3\right\} \setminus \left\{ n,m\right\} ,$ where $a_{j,nq}$ is the element in the *n*th row and *q*th column of the coefficient matrix $A_j.$

**Fig. 8:**
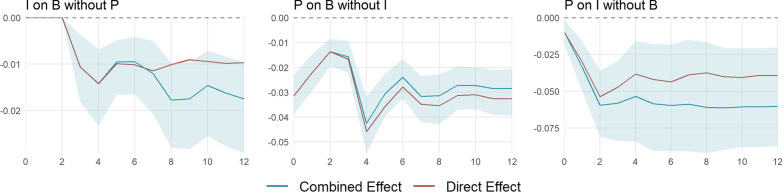
Cumulative impulse responses with indirect channels shut off. The impulse responses are estimated using the recursively ordered VAR(4) in (4) with $k=3$ and $P=\Delta$ ${\text {KSI}}^+$, $B=\Delta \ln {\text {NTRX}}$, and $I = \ln R_e$ with cantonal fixed effects (blue). The red lines represent the cumulative effect when the effect through the third variable in the system is artificially shut off. The data span September 28, 2020, until April 18, 2021. The shaded areas represent the 95% confidence intervals based on a wild bootstrap procedure with 5000 repetitions. The horizontal axes depict the time horizon in weeks. The vertical axes show the level response of $\ln {\text {NTRX}}$ (left and middle) and $\ln {\text {NINF}}$ (right)

Since the effect of behavior *B* on policy *P* is zero by construction, the corresponding IR reflects only indirect effects through infection *I*. Moreover, the IR of *I* on *P* and *B* on *I* do not contain indirect effects through *B* and *P*. Figure 8 shows the remaining direct effects (red) alongside their overall effect counterparts (blue). The reaction of behavior to an infection shock (left panel) is not channeled through changes in the ${\text {KSI}}^+$ broadly until week 7. Thereafter, one third to one half of the reduction in behavior is due to the policy reaction to the infection shock. Hence, the short-term reaction of behavior to rising infections is voluntary, while in the long run, the policy response and the reaction thereto accounts for close to half of the response. Without the infection channel, a policy increase leads to a slightly stronger reduction in consumption starting in week 4, since the negative effect of policy on infection growth does not push up behavior. In contrast, the response of infection growth to a policy increase is weakened when the behavior channel is shut off. Approximately one third of the reduction in the level of infections is due to a decline in the number of debit card transactions.

### Sub-categories of policy (*P*)

The KOF Stringency Indices broadly summarize the stringency of non-pharmaceutical containment measures by taking several indicators into account. Each indicator is equally weighted, and the nuances within the indicators are uniformly assigned. Yet, the effectiveness of different containment measures is likely to vary with its intensity as well as broad category. Analyzing each individual sub-indicator is not feasible for at least four reasons. First, sub-indicators that do not vary across cantons will be absorbed by time fixed effects. Second, limited variation within a sub-indicator over time reduces the possibility to statistically identify any effects. Third, measures were partly introduced simultaneously rendering multicollinearity problems. Fourth, the effectiveness of one measure is likely to depend upon other measures, i.e., the effectiveness of the whole package is likely to be greater than the sum of its parts. Nevertheless, to the extent feasible, we examine the marginal impact of those sub-indicators $\upsilon _{\ell ,t}, \ell \in L$, for which sufficient variation exists. To that end, we estimate (5) for each forecast horizon to obtain Local-Projection Impulse Responses of infection growth to different policy shocks.

To validate the LP-IR method and to establish a benchmark policy effect, we first estimate the local projection equation (5) with the regular ${\text {KSI}}^+$ with and without time fixed effects. The results are shown in the left panel of Fig. 14 in Appendix 4. The impulse responses are similar to their VAR counterparts, especially for the model including time fixed effects. In the combined model, the negative effect is only temporary and turns insignificant in week three. When time fixed effects are included, the drop in infections amounts to $\exp \left( -0.058\cdot 2.5\right) -1=-13.5\%$ after four weeks.

**Fig. 9:**
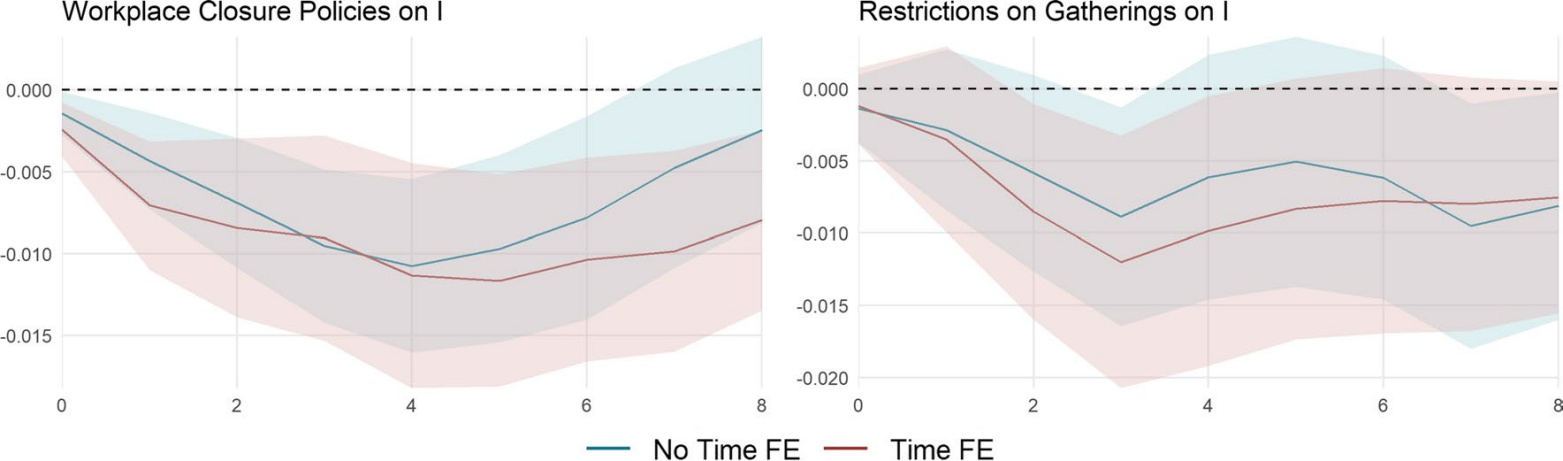
Cumulative impulse responses of infection growth *I*. The impulse responses are estimated using the local projection specification in (5) with $P=\Delta$ ${\text {KSI}}^+$, $B=\Delta \ln {\text {NTRX}}$, and $I = \ln R_e$ with (red) and without (blue) time fixed effects. The data span September 28, 2020, until April 18, 2021. The shaded areas represent the 95% confidence intervals based on standard errors corrected for serial correlation. The horizontal axes depict the time horizon in weeks. The vertical axes show the level response of the respective ${\text {KSI}}^+$ subcategory

All sub-indicators for which there is no time or cantonal variation observed in our sample are not analyzed individually. These are *school closing (c1)*, *close public transport (c5)*, *stay at home requirements (c6)*, *domestic travel (c7)*, *international travel (c8)*, and *public info campaign (h1)* (see Fig. 12 in Appendix 4). The four remaining indicators are shown in Fig. 3. We further exclude *cancel public events (c3)* and *facial coverings (h6)* since, in each case, there is only one increase at the cantonal level, roughly happening at about the same time, making identification factually impossible. For *workplace closing (c2a)* and *restrictions on gatherings (c4)*, the results with (red) and without (blue) time fixed effects are shown in Fig. 9. Note that the sub-categories are normalized such that they lie between 0 and 100.

An increase in *workplace closing (c2a)* significantly reduces infection growth in both models. Four weeks after a 25-unit change in measure *c2a* is enforced, the level of infections drops by roughly $\exp \left( -0.011\cdot 25\right) -1=-24\%$. A 25-unit increase corresponds to an increase by one category within *c2a*, which in turn corresponds to a 2.5 unit increase in the ${\text {KSI}}^+$. Thus, the effect of the sub-category *workplace closing (c2a)* is almost twice as large as an average policy increase, implying a 13.5% reduction in the level of infections. Though estimated with less precision, the effect of the sub-category *restrictions on gatherings (c4)* is similar. Looking at the remaining stringency index ${\text {KSI}}_{-\{c2a,c4\}}^+$ (right panel in Fig. 14 in Appendix 4) reveals the importance of measures related to closures of non-essential businesses and working from home recommendations or requirements. The effect of all other measures turns insignificant.

### Robustness checks

**Fig. 10:**
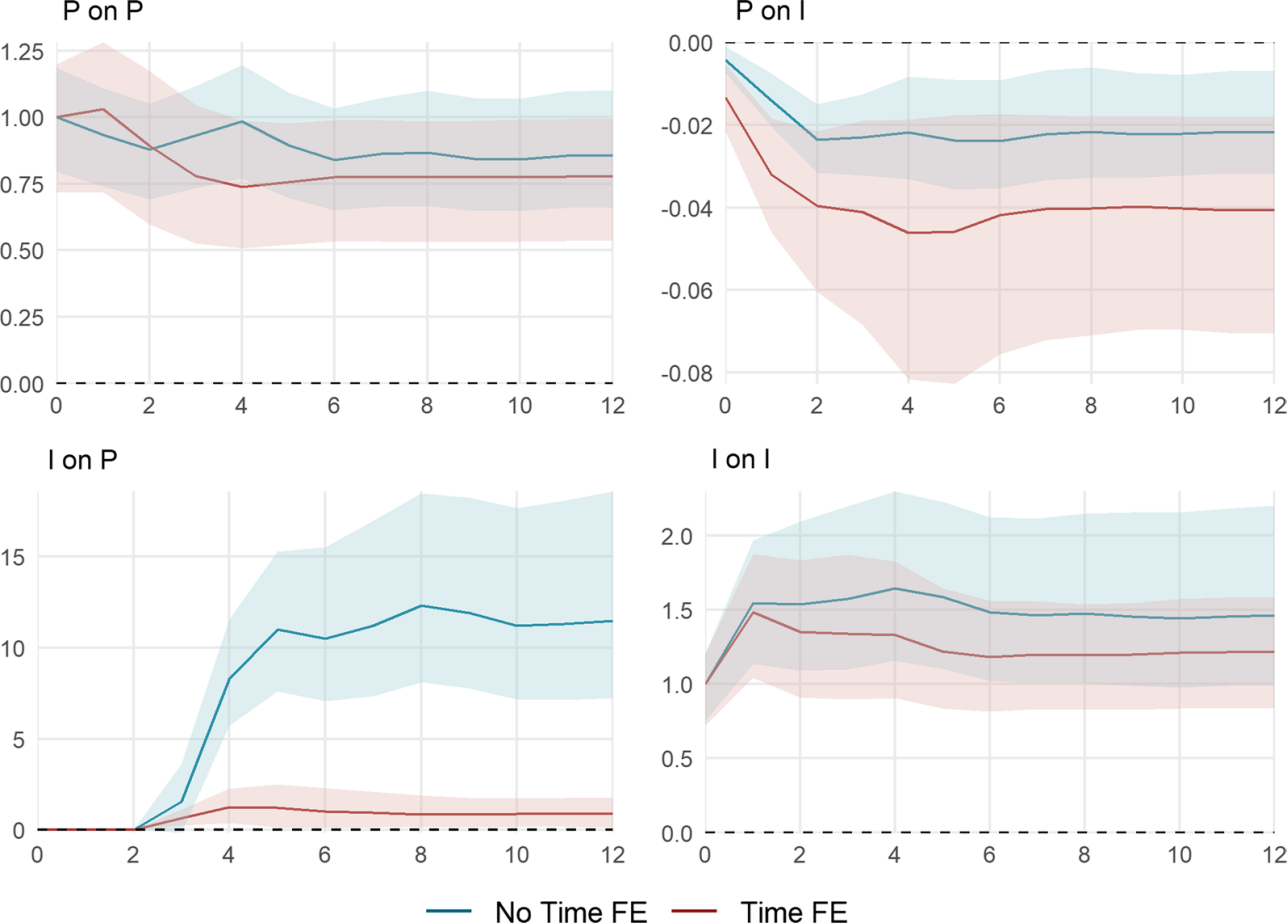
Cumulative impulse responses of policy *P* and infection growth *I*. The impulse responses are estimated using the recursively ordered VAR(4) in (4) with $k=3$ and *B* omitted, $P=\Delta$ ${\text {KSI}}^+$, $I = \ln R_e$ and with (red) and without (blue) time fixed effects. The data span September 28, 2020, until April 18, 2021. The shaded areas represent the 95% confidence intervals based on a wild bootstrap procedure with 5000 repetitions. The horizontal axes depict the time horizon in weeks. The vertical axes show the level response of the ${\text {KSI}}^+$ (left) and $\ln {\text {NINF}}$ (right)

We conduct several robustness checks to validate our findings. Most containment measures target behavioral changes that in turn lower infection growth. The correlation of our policy variable $P=\Delta$ ${\text {KSI}}^+$ and behavior variable $B=\Delta \ln {\text {NTRX}}$ amounts to − 0.41 in the estimation sample. To address possible multicollinearity concerns, we estimate a second VAR omitting behavior. The results do not differ from those with behavior (see Fig. 10).

**Fig. 11:**
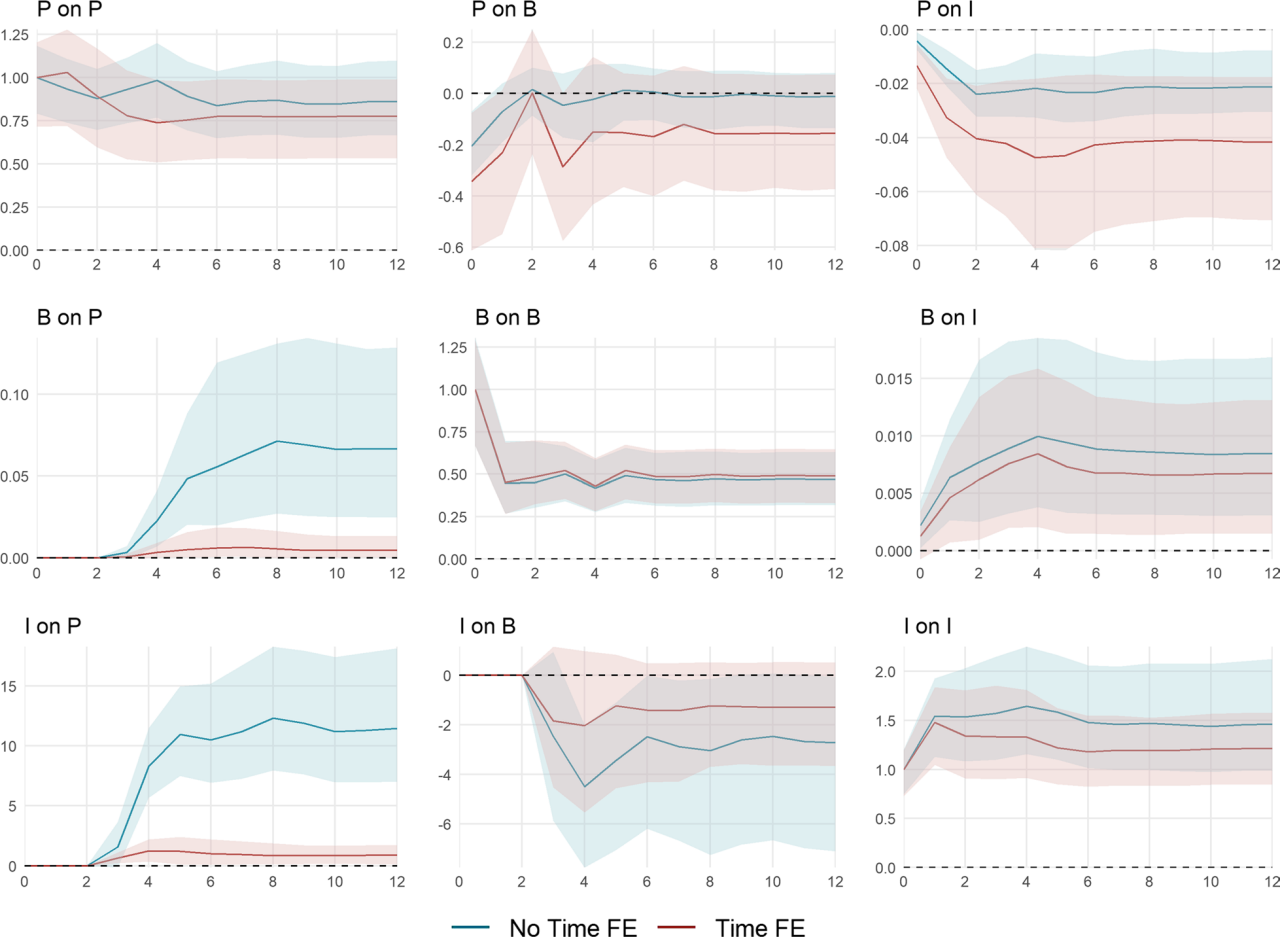
Cumulative impulse responses of policy *P*, behavior (mobility) *B*, and infection growth *I*. The impulse responses are estimated using the recursively ordered VAR(4) in (4) with $k=3$ and $P=\Delta$ ${\text {KSI}}^+$, $B=\Delta dist$, and $I = \ln R_e$ with (red) and without (blue) time fixed effects. The data span September 28, 2020, until April 18, 2021. The shaded areas represent the 95% confidence intervals based on a wild bootstrap procedure with 5000 repetitions. The horizontal axes depict the time horizon in weeks. The vertical axes show the level response of the ${\text {KSI}}^+$ (left), *dist* (middle) and $\ln {\text {NINF}}$ (right)

**Fig. 12:**
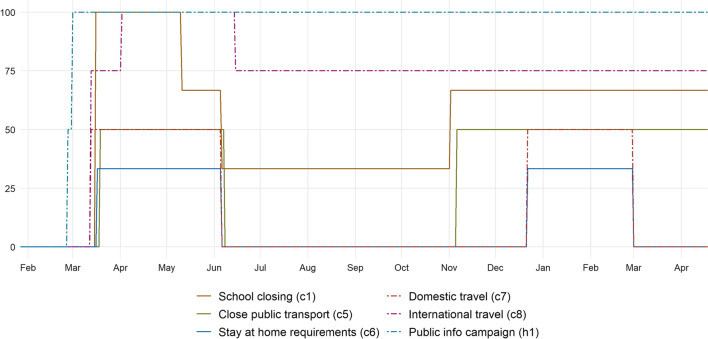
Sub-categories of the ${\text {KSI}}^+$ without cantonal variation. Graph shows the sub-indicators of the KOF Stringency-Plus Index that exhibit no cantonal variation. The respective sub-indicator is denoted on the *y*-axis. Note that cantonal variation only starts end of June 2020. The first lockdown was governed by federal measures

**Fig. 14:**
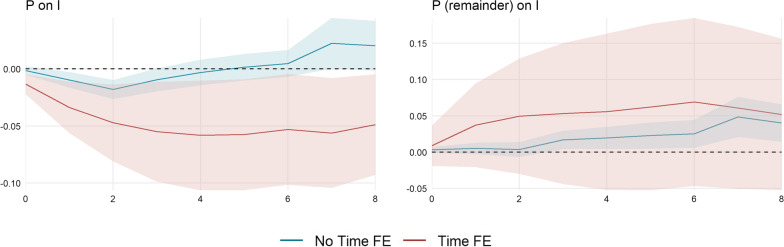
LP cumulative impulse responses of infection growth *I*. The impulse responses are estimated using the local projection specification in (5) with $p=4$, $P=\Delta$ ${\text {KSI}}^+$ (left), $P_{\{c2a,c4\}}=\Delta$ ${\text {KSI}}$ $_{-\{c2a,c4\}}^+$ (right), $B=\Delta \ln {\text {NTRX}}$, and $I = \ln R_e$ with (red) and without (blue) time fixed effects. In the left panel, the sub-categories are omitted and in the right panel, the aggregate policy index does exclude Workplace Closings (c2a) and Restrictions on Gatherings (c4) since they enter the equation individually. The data span June 22, 2020, until January 17, 2021. The shaded areas represent the 95% and 90% confidence intervals based on standard errors corrected for serial correlation. The horizontal axes depict the time horizon in weeks. The vertical axes show the level response of $\ln {\text {NINF}}$

Consumer spending captures only one specific aspect of behavior in response to changes in NPIs and infections. In a next step, we use the median distance travelled in kilometers to approximate behavior. Fewer debit transactions go hand in hand with reduced mobility and, thus, the general relations in the VAR should remain valid. The results with $B = \Delta dist$ are shown in Fig. 11. The effects between policy *P* and infection growth *I* remain unchanged. The impulse responses involving mobility are qualitatively similar, though less precisely estimated. A one-unit policy increase decreases the median distance by 0.21 km to 0.34 km on impact. For both models, the effect turns insignificant for all other horizons. A one-kilometer shock to the median distance traveled leads to an $\exp \{0.01\}-1=1.0\%$ increase in the level of weekly infections after four weeks. The marginal effect after four weeks is 0.8%, though not statistically different from the combined effect. A 10% increase in weekly infections implies a permanent reduction in mobility of $-4.5\ln (1.1)=-0.43$ km after four weeks. Again, the marginal cantonal effect is less pronounced but neither statistically significant nor different from the combined effect.

**Fig. 15:**
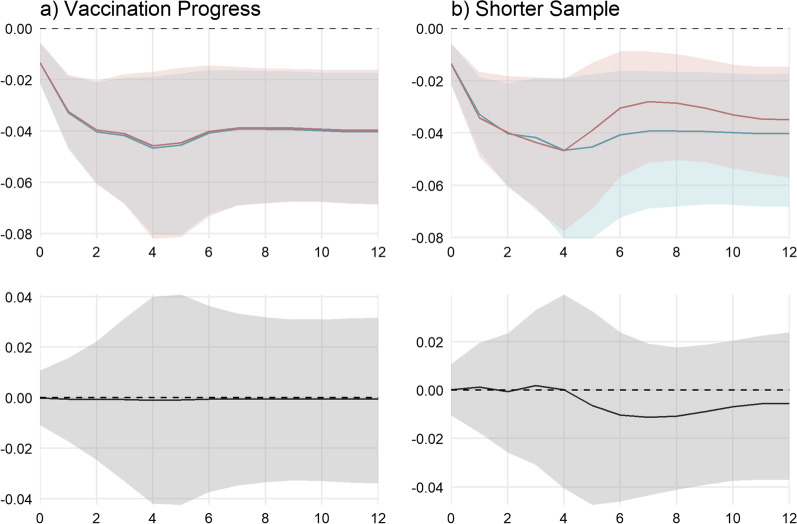
Cumulative impulse responses of infection growth *I* to policy *P* for two alternative models. The impulse responses are estimated using the recursively ordered VAR(4) in (4) with $k=3$ and $P=\Delta$ ${\text {KSI}}^+$, $B=\Delta \ln {\text {NTRX}}$, and $I = \ln R_e$ with canton and time fixed effects. The top panel shows the cumulative IRs of Infection Growth *I* to Policy *P* of the baseline model (blue) and the alternative specification (red). The bottom panel shows the difference between the baseline and alternative IRs. The alternative models are characterized by: **a** two additional control variables concerning vaccination, namely the (lagged) change in the number of fully as well as partially vaccinated residents (left), and **b** a shorter sample length (right). Both variants do not include the interaction terms on vaccination progress. The data span from September 28, 2020, until April 18, 2021 for model (**a**), and until January 17, 2021 for model (**b**). The shaded areas represent 95% confidence intervals based on a wild bootstrap procedure with 5000 repetitions. The horizontal axes depict the time horizon in weeks. The vertical axes are shown in units of $\ln {\text {NINF}}$

During Phase 4, two factors emerged that potentially distort our findings. First, the progress of the vaccination program gradually reduces the susceptible population. To address this, our baseline models include interaction terms between the cantonal shares of the population being 65 years and older and the number of newly partially and fully vaccinated residents (nationwide). Second, the so-called UK-mutation B.1.1.7, or Alpha, a variant of SARS-CoV-2 that is clearly more transmissible than the wild-type, became more and more prevalent in Switzerland (Davies et al., 2021). To check whether these aspects affect our results, we estimate two alternatives to our baseline model in (4) with time fixed effects and without the interaction terms regarding vaccination progress. First, we add two cantonal vaccination variables: the change in the number of fully and partially vaccinated residents provided by the Swiss Federal Office of Public Health (FOPH). Although there is a risk that vaccination progress at the cantonal level is endogenous, we believe that this problem is probably not that severe, since the distribution of vaccine supplies by the army pharmacy in the early stages of the vaccination campaign was based on population shares only.^17^ Second, we end our estimation window on January 17, when neither vaccination campaign nor the prevalence of variant B.1.1.7 (Alpha) were well-advanced. The results are shown in Fig. 15 in Appendix 4. None of the model specifications changes the main results.^18^

## Conclusion

In this paper, we study the interplay between non-pharmaceutical containment measures, the spread of COVID-19, and public behavior in Switzerland. First, we construct cantonal indices to proxy the stringency of COVID-19 containment measures. In a second step, we employ a vector autoregressive (VAR) framework to estimate impulse response functions of three endogenous variables: the effective reproductive number, human behavior measured by debit card transactions, and containment measures imposed by governments as quantified by the KOF Stringency-Plus Index.

Our study focuses on two phases from September 28, 2020, to April 18, 2021. During the first phase, cantonal governments where able to set policies in accordance with their regional epidemiological situation. In contrast, the second phase is characterized by federal measures that apply to all cantons equally. In our analysis, we differentiate between overall and canton-specific effects by introducing time fixed effects. In the model without time fixed effects, federal as well as cantonal changes are included, with the disadvantage that any other time-specific effect not captured by one of our variables might introduce a bias in our coefficient estimates. However, when adding time fixed effects to the model, federal shocks are fully absorbed and the results represent only canton-specific effects.

The results indicate that an increase in the stringency of non-pharmaceutical measures induces significant and sizable reductions in infection growth. A 10-unit increase in policy stringency results in a 34% reduction in weekly infections after six weeks. Further, a policy shock leads to a decrease in debit card transactions. This indicates that stricter federal measures actually led to behavioral changes in the population. Conversely, a rise in infection growth induces policy reactions in form of stricter containment measures by federal and cantonal governments. Similar to the policy shocks, debit card transactions decrease in response to an infection shock. In fact, behavioral changes are voluntary in the short term while half of the long-run changes are attributed to stricter policies. In line with Hacıoğlu-Hoke et al. (2021) and Gupta et al. (2020), the drop in consumer spending and mobility precedes the introduction of new policy measures. When considering different measures individually, we find that workplace and business closings as well as restrictions on gatherings are particularly effective in containing the spread of COVID-19.

Our analysis has relevant policy implications. First, we show that non-pharmaceutical containment measures helped combat the COVID-19 pandemic. Hence, implementing restrictions can significantly reduce the spread of a viral epidemic. Second, voluntary behavioral adaptations played a non-negligible role in reducing the spread on top of mandatory restrictions set by federal and cantonal governments, which amplifies the effects of potential policies. Third, closings of workplaces and restrictions on gatherings were very effective in containing the spread. This should be taken into account in future fights against pandemics.

## Figures and Tables

**Fig. 13:**
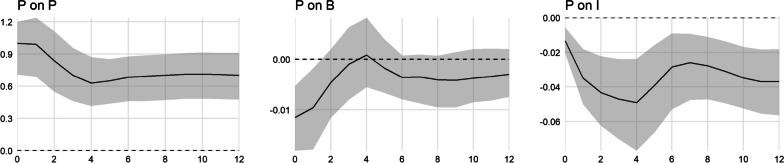
Cumulative impulse responses of policy *P*, behavior (consumption) *B*, and infection growth *I* to a unit policy shock (only Phase 3). The impulse responses are estimated using the recursively ordered VAR(4) in (4) with $k=3$ and $P=\Delta$ ${\text {KSI}}^+$, $B=\Delta \ln {\text {NTRX}}$, and $I = \ln R_e$ with time fixed effects. The data span September 28, 2020, until January 17, 2021. The shaded areas represent the 95% confidence intervals based on a wild bootstrap procedure with 5000 repetitions. The horizontal axes depict the time horizon in weeks. The vertical axes show the level response of the ${\text {KSI}}^+$ (left), $\ln {\text {NTRX}}$ (middle), $\ln {\text {NINF}}$ (right)

## Data Availability

All data are publicly available, except the data on mobility.

## Appendix 1: Sources for stringency indices

All websites were last accessed: June 29, 2021.

Oxford COVID-19 Government Response Tracker:

https://github.com/OxCGRT/covid-policy-tracker/blob/master/documentation/codebook.md (Codebook)

https://github.com/OxCGRT/covid-policy-tracker/blob/master/documentation/index_methodology.md (Methodology)

Federal Office of Public Health (FOPH) Website for national measures:

https://www.bag.admin.ch/bag/de/home/krankheiten/ausbrueche-epidemien-pandemien/aktuelle-ausbrueche-epidemien/novel-cov/massnahmen-des-bundes.html

Cantonal Health Association (GDK) Website for cantonal measures:

https://www.gdk-cds.ch/de/praevention-und-gesundheitsfoerderung/neues-coronavirus

State Secretariat for Migration (SEM) for international travel:

https://www.sem.admin.ch/sem/de/home/sem/aktuell/faq-einreiseverweigerung.html

Information on public campaigns:

https://www.bag.admin.ch/bag/de/home/das-bag/aktuell/medienmitteilungen.msg-id-78273.html

https://de.wikipedia.org/wiki/COVID-19-Pandemie_in_der_Schweiz#Februar_2020

Information on public transport:

https://company.sbb.ch/de/medien/medienstelle/medienmitteilungen/detail.html/2020/3/1803-1

https://news.sbb.ch/artikel/95719/die-neusten-informationen-zum-coronavirus-2-4-2020

https://news.sbb.ch/artikel/95750/coronavirus-diese-schutzmassnahmen-machen-reisen-moeglichst-sicher

Additional information from ETH-Council and the Federal Office of Public Health.

## Appendix 2: Bootstrap procedure

Let $\left( \mathbf {y_i}, \mathbf {X_i}\right) , i=1,\ldots , n$ be the original sample, where $\mathbf {y_i}$ is a $T\times 1$ vector and $\mathbf {X_i}$ a $T\times n$ matrix. Furthermore, let $\mathbf {{\hat{\beta }}}$ be a $T\times n$ matrix with the corresponding coefficient estimates and by $\mathbf {{\hat{\epsilon }}_i}$ we denote the $T\times 1$ residual vectors.

For the wild cluster bootstrap procedure (see, e.g., Cameron et al., 2008), we create *B* pseudo-samples $\mathbf {{\hat{\epsilon }}_i^*} = a_i {\hat{\epsilon _i}}, i =1,\ldots ,n$ using the weights $a_i = \frac {(1-\sqrt{5})}{2}$ with probability $\frac {(1+\sqrt{5})}{(2\sqrt{5})}$ and $a_i = \frac {(1+\sqrt{5})}{2}$ with probability $1-\frac {(1+\sqrt{5})}{(2\sqrt{5})}$, as suggested by Mammen (1993). The weights $a_i$ have zero mean, unit variance and ${\text {E}}\left[ a_i^3\right] =1.$ We then form $\left( \mathbf {y_i^*}, \mathbf {X_i}\right)$ to obtain ${{\hat{\beta }}^*}$. The resulting sample ${{\hat{\beta }}^*}_1,\ldots , {{\hat{\beta }}^*}_B$ is then used to form statistical inference.

## Appendix 3: Interpretation of $R_e$

The effective reproduction number represents the ratio between the number of new infections (NINF) on day *t* and the infectious population prior to that:

$$\begin{aligned} R_e(t)&= \frac{{\text {NINF}}_t}{\sum _{s=1}^t {\text {NINF}}_{t-s}w_s}, \end{aligned}$$

where $w_s$ is the value of the infectivity profile *s* days after infection, $\sum _s w_s = 1$, modelled by the serial interval distribution (Huisman et al., 2020). For simplicity, assume $w_s = \mathbbm {1}_{\{s=\tau \}}$, i.e., transmission only takes place exactly $\tau$ days after infection. Then,

$$\begin{aligned} R_e(t) = \frac{{\text {NINF}}_t}{{\text {NINF}}_{t-\tau }} \end{aligned}$$

and $\ln R_e$ describes the $\tau$-day growth rate of the number of new infections. This implies that

$$\begin{aligned} \sum _{k=1}^h \ln R_{e, t+k}&= {\left\{ \begin{array}{ll} \sum _{k=0}^{\tau -1} \ln {\text {NINF}}_{t+h-k} - \sum _{k=0}^{\tau -1} \ln {\text {NINF}}_{t-1-k} &{} h \ge \tau ,\\ \sum _{k=0}^{h} \ln {\text {NINF}}_{t+h-k} - \sum _{k=0}^{h} \ln {\text {NINF}}_{t+h-k-\tau } &{} h<\tau .\end{array}\right. } \end{aligned}$$

For $h\ge \tau$, the estimated IR thus indicate the log level effect between the sum of new infections in $\{h-\tau +1,\ldots ,h\}$ and that in $\{t-\tau ,\ldots ,t-1\}$. For $h<\tau ,$ we obtain the log level change between the sum of new infections in $\{t,\ldots ,t+h\}$ and that in $\{t-\tau , \ldots , t+h-\tau \}$.

## Appendix 4

See Figs. 12, 13, 14, 15.

## Appendix 5

See Tables 2 and 3.

**Table 2:** VAR(3) with canton fixed effects

	Dependent variable		
	$\Delta$ KOF Stringency-Plus Index	$\Delta \ln$ Number of debit transactions	ln effective reproductive number
$\Delta$ KOF Stringency-Plus Index (lag 1)	− 0.067*	0.0004	− 0.006***
	(0.037)	(0.001)	(0.002)
$\Delta$ KOF Stringency-Plus Index (lag 2)	− 0.058*	0.0002	− 0.006***
	(0.033)	(0.001)	(0.001)
$\Delta$ KOF Stringency-Plus Index (lag 3)	0.051	0.001	0.005***
	(0.034)	(0.001)	(0.002)
$\Delta$ KOF Stringency-Plus Index (lag 4)	0.098**	− 0.011***	0.001
	(0.041)	(0.001)	(0.002)
$\Delta \ln$ number of debit transactions (lag 1)		− 0.262***	0.109**
		(0.041)	(0.055)
$\Delta \ln$ number of debit transactions (lag 2)		− 0.329***	0.048
		(0.040)	(0.054)
$\Delta \ln$ number of debit transactions (lag 3)		0.009	0.200***
		(0.039)	(0.053)
$\Delta \ln$ number of debit transactions (lag 4)		− 0.070*	0.114**
		(0.041)	(0.055)
ln Effective reproductive number (lag 1)			0.544***
			(0.041)
ln effective reproductive number (lag 2)			− 0.322***
			(0.044)
ln effective reproductive number (lag 3)	1.565*	− 0.103***	0.212***
	(0.935)	(0.028)	(0.045)
ln effective reproductive number (lag 4)	5.969***	− 0.006	− 0.054
	(0.808)	(0.024)	(0.033)
Public holiday	5.806***	− 0.462***	0.141*
	(1.845)	(0.056)	(0.074)
School holiday	1.395***	− 0.090***	− 0.002
	(0.317)	(0.010)	(0.013)
$\Delta$ maximum temperature (in °C)	0.111***	0.002*	− 0.001
	(0.029)	(0.001)	(0.001)
$\Delta$ precipitation (in mm)	− 0.061**	− 0.001	0.002
	(0.028)	(0.001)	(0.001)
$\Delta$ sunshine hours	0.025	0.001	− 0.004
	(0.072)	(0.002)	(0.003)
$\Delta$ mean relative humidity (in %)	− 0.065***	0.0005	− 0.001
	(0.020)	(0.001)	(0.001)
$\Delta$ share65 $\cdot$ persons fully vaccinated (CH)	− 0.629***	0.004*	0.012***
	(0.077)	(0.002)	(0.003)
$\Delta$ share65 $\cdot$ persons partially vaccinated (CH)	0.004	− 0.007***	− 0.004**
	(0.045)	(0.002)	(0.002)
Observations	650	650	650
R$^{2}$	0.456	0.484	0.496
Adjusted R$^{2}$	0.422	0.447	0.459
Residual std. error	2.646 (*df* = 610)	0.078 (*df* = 606)	0.104 (*df* = 604)
*F* statistic	13.135*** (*df* = 39; 610)	13.209*** (*df* = 43; 606)	13.227*** (*df* = 45; 604)

The model specification is given in (4) with canton fixed effects and four weeks of lags ($p=4$), $P=\Delta$ ${\text {KSI}}^+$, $B=\Delta \ln {\text {NTRX}}$, and $I = \ln R_e$. The sample includes weekly data from September 28, 2020 until April 18, 2021 for each of the 26 cantons. $\Delta$ denotes the first difference operator and ln the natural logarithm. *$p<0.1$; **$p<0.05$; ***$p<0.01$

**Table 3:** VAR(3) with canton and time fixed effects

	Dependent variable		
	$\Delta$ KOF Stringency-Plus Index	$\Delta \ln$ number of debit transactions	ln effective reproductive number
$\Delta$ KOF Stringency-Plus Index (lag 1)	0.030	0.001	− 0.012***
	(0.032)	(0.003)	(0.004)
$\Delta$ KOF Stringency-Plus Index (lag 2)	− 0.139***	0.004	− 0.003
	(0.029)	(0.002)	(0.004)
$\Delta$ KOF Stringency-Plus Index (lag 3)	− 0.096***	0.002	− 0.004
	(0.028)	(0.002)	(0.004)
$\Delta$ KOF Stringency-Plus Index (lag 4)	− 0.038	0.0003	− 0.006
	(0.028)	(0.002)	(0.004)
$\Delta \ln$ number of debit transactions (lag 1)		− 0.170***	0.132**
		(0.037)	(0.066)
$\Delta \ln$ number of debit transactions (lag 2)		− 0.196***	0.044
		(0.036)	(0.065)
$\Delta \ln$ number of debit transactions (lag 3)		− 0.160***	0.079
		(0.036)	(0.064)
$\Delta \ln$ number of debit transactions (lag 4)		− 0.209***	0.071
		(0.036)	(0.064)
ln effective reproductive number (lag 1)			0.477***
			(0.042)
ln effective reproductive number (lag 2)			− 0.368***
			(0.046)
ln effective reproductive number (lag 3)	0.665**	− 0.083***	0.229***
	(0.284)	(0.022)	(0.045)
ln effective reproductive number (lag 4)	0.249	− 0.014	− 0.140***
	(0.279)	(0.022)	(0.041)
Public holiday	− 0.735	− 0.452***	− 0.151
	(0.909)	(0.072)	(0.127)
School holiday	− 0.056	− 0.041***	0.030*
	(0.110)	(0.009)	(0.015)
$\Delta$ maximum temperature (in °C)	− 0.028	− 0.001	− 0.003
	(0.030)	(0.002)	(0.004)
$\Delta$ precipitation (in mm)	− 0.007	− 0.006***	− 0.001
	(0.013)	(0.001)	(0.002)
$\Delta$ sunshine hours	− 0.026	− 0.002	− 0.003
	(0.029)	(0.002)	(0.004)
$\Delta$ mean relative humidity (in %)	− 0.012	− 0.001**	− 0.001
	(0.007)	(0.001)	(0.001)
$\Delta$ share65 $\cdot$ persons fully vaccinated (CH)	− 0.027	− 0.012	− 0.007
	(0.142)	(0.011)	(0.020)
$\Delta$ share65 $\cdot$ persons partially vaccinated (CH)	− 0.049	− 0.017*	− 0.036**
	(0.117)	(0.009)	(0.016)
Observations	650	650	650
$R^{2}$	0.963	0.749	0.572
Adjusted $R^{2}$	0.959	0.720	0.521
Residual std. error	0.707 (*df* = 586)	0.056 (*df* = 582)	0.098 (*df* = 580)
*F* statistic	240.025*** (*df* = 63; 586)	25.869*** (*df* = 67; 582)	11.214*** (*df* = 69; 580)

The model specification is given in (4) with canton and time fixed effects and four weeks of lags ($p=4$), $P=\Delta$ ${\text {KSI}}^+$, $B=\Delta \ln {\text {NTRX}}$, and $I = \ln R_e$. The sample includes weekly data from September 28, 2020 until April 18, 2021 for each of the 26 cantons. $\Delta$ denotes the first difference operator and ln the natural logarithm

*$$p<0.1$$; **$$p<0.05$$; ***$$p<0.01$$

## Abbreviations

AI: Appenzell–Innerrhoden
AR: Appenzell–Ausserrhoden
ATM: Automated teller machine
B: Behavior (changes)
BL: Basel–Landschaft
BS: Basel–Stadt
FDHA: Swiss Federal Department of Home Affairs
FE: Fixed effects
FOPH: Swiss Federal Office of Public Health
GDK: Cantonal Health Association
HPDR: Highest posterior density range
I: Infections (growth)
IR: Impulse responses
${\text {KSI}}$: KOF Stringency Index
${\text {KSI}}^+$: KOF Stringency-Plus Index
LOESS: Local polynomial regression fitting
LP: Local projections
MA: Moving average
NINF: Number of new infections
NPI: Non-pharmaceutical intervention
NTRX: Number of debit card transactions
NW: Nidwalden
OW: Obwalden
P: Policy (changes)
$R_e$: Effective reproductive number
SEM: State Secretariat for Migration
SFSO: Swiss Federal Statistical Office
SG: St. Gallen
SMN: SwissMetNet
VAR: Vector autoregressive model
VS: Valais

## Footnotes

1 The data are available here: https://kof.ethz.ch/prognosen-indikatoren/indikatoren/kof-stringency-index.html.

2 The extraordinary situation is according to Article 7 of the Epidemics Act declaratory in nature and reaffirms the Federal Council’s constitutional competence at the legislative level to make use of the right of emergency (according to Article 185 paragraph 3 of the Federal Constitution). The constitutional right of emergency allows the Federal Council to order appropriate measures quickly and on a case-by-case basis in the event of unforeseeable, acute and serious threats to public health that may endanger the internal security of the country.

3 Note that Equation (5) contains *B* and *P* contemporaneously such that structural impulse responses are directly obtained.

4 On December 11, 2020, the Swiss Federal Council decided that cantons may extend opening hours to 11pm if their epidemiological situation allows for that. Further, a decision on December 18, 2020, enabled cantons with a favorable epidemiological situation to relax certain restrictions, such as closures of restaurants and sports facilities.

5 Detailed information on the coding of the sub-indicators is provided here: https://github.com/OxCGRT/covid-policy-tracker/blob/master/documentation/codebook.md, last accessed January 11, 2021.

6 The resulting categories are: 0—No measures; 1—Recommend closing (or work from home); 2—Reduction in opening hours and/or capacity ; 3—Require closing (or work from home) for some sectors or categories of workers; 4—Require closing (or work from home) all-but-essential workplaces (e.g., grocery stores, doctors).

7 In particular, they use first-order polynomials and tricubic weights and, for each point in time, a window of 21 days.

8 They extend the method by Goldstein et al. (2009) to handle time varying delay distributions and missing data by using line list data on the delay between the onset of symptoms and a reported case, provided by the Federal Office of Public Health (FOPH), to estimate time-varying delay distributions between transmission and reporting of cases, hospitalizations, and deaths. The delay between transmission and confirmation by a positive test result depends on the availability of tests and the time between test and test result. Similarly, the delay between hospital transmission and or death depends on the health characteristics of the population and hospital capacities and availability. Hence, the resulting delay distributions reflect characteristics of the Swiss population, its health care system and changes in capacities related to testing and tracing.

9 The resulting time series of $R_e$ is publicly available: https://github.com/covid-19-Re/dailyRe-Data, last accessed on May 26, 2021.

10 Monitoring Consumption Switzerland is a joint project by the University of St. Gallen and the University of Lausanne, see https://monitoringconsumption.com.

11 The data on the number of transactions do not include e-commerce.

12 Some hypothesize that COVID-19 contagiousness is promoted by high levels of particulate matter and thereby weather conditions. See, e.g., Zhu et al. (2020), Fattorini and Regoli (2020), Zoran et al. (2020), Li et al. (2021), Wu et al. (2020).

13 The results are robust to different lag specifications. Given the publication delay, we have experimented with lags $p\ge 3$.

14 More generally, let $y_1=\Delta \ln z_1$ and $y_2 = \Delta z_2$, i.e., $z_1$ enters the model in log differences and $z_2$ in first differences (for instance $y_1= I, z_1 = {\text {NINF}}, y_2 = P, z_2 =$ ${\text {KSI}}^+$). Then, a shock to $y_1$ of size *s* changes $\ln z_1$ by *s* points and $z_1$ by $100\cdot \left( \exp \left\{ s\right\} -1\right) = p$ percent on impact. Let ${\text {IR}}_h^{cum}$ be the cumulative impulse response of $y_2$ to a normalized shock to $y_1$ after *h* periods ($s=1$). Then, an *s* point shock to $\ln z_1$ or equivalently a *p* percentage change in $z_1$ changes $z_2$ by $s \cdot {\text {IR}}_h^{cum}$ points. On the other hand, a shock to $y_2$ of size *s* changes $z_2$ by *s* points on impact. An *s* shock changes $\ln z_1$ by $s \cdot {\text {IR}}_h^{cum}$ and $z_1$ by $100\cdot \left( \exp \left\{ s \cdot {\text {IR}}_h^{cum}\right\} -1\right) = p$ percent. If instead, $y_2 = \Delta \ln z_2$, i.e., $z_2$ also enters in log-differences, an *s* shock to $\ln z_1$ or *p* percentage point change in $z_1$ leads to a $100 \exp \left\{ s \cdot {\text {IR}}_h^{cum}\right\} = 100\cdot \exp \left\{ \ln \left( \frac {100+p}{100}\right) \cdot {\text {IR}}_h^{cum}\right\} = \left( \frac {100+p}{100}\right) ^{{\text {IR}}_h^{cum}}$ percent change.

15 To address the concern of confounding factors with respect to the incidence level, we have included it in $x_{i,t}$. The impulse responses between the endogenous variables remain unchanged.

16 When time fixed effects are included, all policy changes on the federal level are absorbed. Since Phase 4 exclusively consists of federal policy measures, we effectively estimate the marginal effect of policy during Phase 3. We have repeated the estimation using only Phase 3. The resulting policy effects are in line with our previous findings (see Fig. 13 in Appendix 4).

17 For four cantons (AG, OW, SG, VS), we use averages of the remaining cantons as no data are available.

18 The same applies when conducting both robustness checks for the model without time fixed effects.
